# Therapeutic Approaches to Nonalcoholic Fatty Liver Disease: Exercise Intervention and Related Mechanisms

**DOI:** 10.3389/fendo.2018.00588

**Published:** 2018-10-15

**Authors:** Hirokazu Takahashi, Kazuhiko Kotani, Kenichi Tanaka, Yuichiro Egucih, Keizo Anzai

**Affiliations:** ^1^Division of Metabolism and Endocrinology, Faculty of Medicine, Saga University, Saga, Japan; ^2^Liver Center, Saga University Hospital, Saga University, Saga, Japan; ^3^Division of Community and Family Medicine, Center for Community Medicine, Jichi Medical University, Shimotsuke, Japan

**Keywords:** lifestyle modification, exercise protocol, training protocol, organ crosstalk, hepatokines, biomarkers

## Abstract

Exercise training ameliorates nonalcoholic fatty liver disease (NAFLD) as well as obesity and metabolic syndrome. Although it is difficult to eliminate the effects of body weight reduction and increased energy expenditure—some pleiotropic effects of exercise training—a number of studies involving either aerobic exercise training or resistance training programs showed ameliorations in NAFLD that are independent of the improvements in obesity and insulin resistance. *In vivo* studies have identified effects of exercise training on the liver, which may help to explain the “direct” or “independent” effect of exercise training on NAFLD. Exercise training increases peroxisome proliferator-activated receptor gamma coactivator 1-alpha (PGC1α) expression, improves mitochondrial function and leads to reduced hepatic steatosis, inflammation, fibrosis, and tumor genesis. Crosstalk between the liver and adipose tissue, skeletal muscle and the microbiome is also a possible mechanism for the effect of exercise training on NAFLD. Although numerous studies have reported benefits of exercise training on NAFLD, the optimal duration and intensity of exercise for the prevention or treatment of NAFLD have not been established. Maintaining adherence of patients with NAFLD to exercise training regimes is another issue to be resolved. The use of comprehensive analytical approaches to identify biomarkers such as hepatokines that specifically reflect the effect of exercise training on liver functions might help to monitor the effect of exercise on NAFLD, and thereby improve adherence of these patients to exercise training. Exercise training is a robust approach for alleviating the pathogenesis of NAFLD, although further clinical and experimental studies are required.

## Introduction

Nonalcoholic fatty liver disease (NAFLD) is a chronic liver disease related to obesity and one of the manifestations of metabolic syndrome. In accordance with the worldwide pandemic of obesity, NAFLD is considered to be increasing and the global prevalence is estimated as 25.24% (1). A recent systematic review indicated that physical activity and inactivity are associated with all-cause mortality, and high levels of moderate intensity physical activity eliminate the increased risk of death associated with prolonged sitting times (2). Moreover, a number of epidemiological studies have demonstrated a strong correlation between physical activity and non-communicable diseases including diabetes, metabolic syndrome, cardiovascular diseases and cancer (3–6). The prevalence of NAFLD is also related to physical activity. Sitting time was positively correlated with NAFLD prevalence as diagnosed by ultrasonography, independent of body mass index (BMI), in a large cross-sectional study (7). A recent longitudinal epidemiological study involving 169,347 men and women showed a strong negative correlation between habitual exercise and the development of a fatty liver diagnosed by ultrasonography (8). Therefore, exercise is thought to be a safe and economic choice as a therapeutic or preventative strategy against NAFLD. Indeed, numerous clinical trials have demonstrated the efficacy of exercise. However, the independence of any exercise effect on weight loss remains to be determined, and the molecular mechanism for the effect of exercise on ameliorating the pathogenesis of NAFLD is also not wholly understood. In this review, clinical evidence is analyzed in a systematic review manner and experimental evidence is summarized narratively to evaluate the therapeutic effects and mechanisms of exercise training on NAFLD. Note that the definition of “training” in this review is similar to “endurance exercise” and refers to “the number of exercise sessions” over weeks or months. “Exercise” refers to a single bout of exercise. “Exercise training” is used to generalize both exercise and training.

## Exercise training effect on NAFLD in clinical trials: a systematic review

### Method

A published literature search was conducted in the PubMed, Web of Science, and Scopas databases to December 31, 2017. The following search terms were used to identify the relevant articles: non-alcoholic steatohepatitis OR non-alcoholic fatty liver OR fatty liver OR liver steatosis OR NAFLD OR NASH; exercise OR training. Two readers independently (H.T and K.T) reviewed the titles and abstracts of selected articles for the determination of inclusion as well as the full texts of selected studies. All relevant abstracts and full-text peer reviewed articles published in English were collected for analysis according to the Preferred Reporting Items for Systematic Reviews and Meta-Analyses statement for the conduct of meta-analyses of observational studies (http://www.prisma-statement.org/). Articles were selected if they met the following inclusion criteria. (i) Study design: randomized controlled trial, non-randomized controlled clinical trial, before and after clinical trial, or observational cohort study. (ii) Study issue: the effects of therapeutic exercise on hepatic steatosis in patients with NAFLD. (iii) Study subjects: patients with NAFLD diagnosed by liver biopsy or abdominal imaging including ultrasonography, computed tomography, and magnetic resonance (MR) imaging. Studies were excluded if they: (i) were not original research reports (systematic reviews, narrative reviews, commentaries, or editorials); (ii) were case reports or conference abstracts; (iii) did not provide sufficient data for this study; (iv) were animal studies; or (v) were in the non-English literature. Finally, 34 clinical studies were selected, and 39 exercise protocols were tested for their efficacy in ameliorating liver steatosis in cases of NAFLD [(9–36), Supplementary Material 1]. Spearman's rank correlation coefficient was used to test any correlations between changes in liver steatosis evaluated with ^1^H magnetic resonance (^1^HMR) and changes in BMI or training related-parameters. Wilcoxon's rank sum test was used to compare protocols with and without dietary consultation.

## Results and discussion

### Clinical question 1. is the exercise training effect on NAFLD independent of body weight reduction?

**Figure 1:**
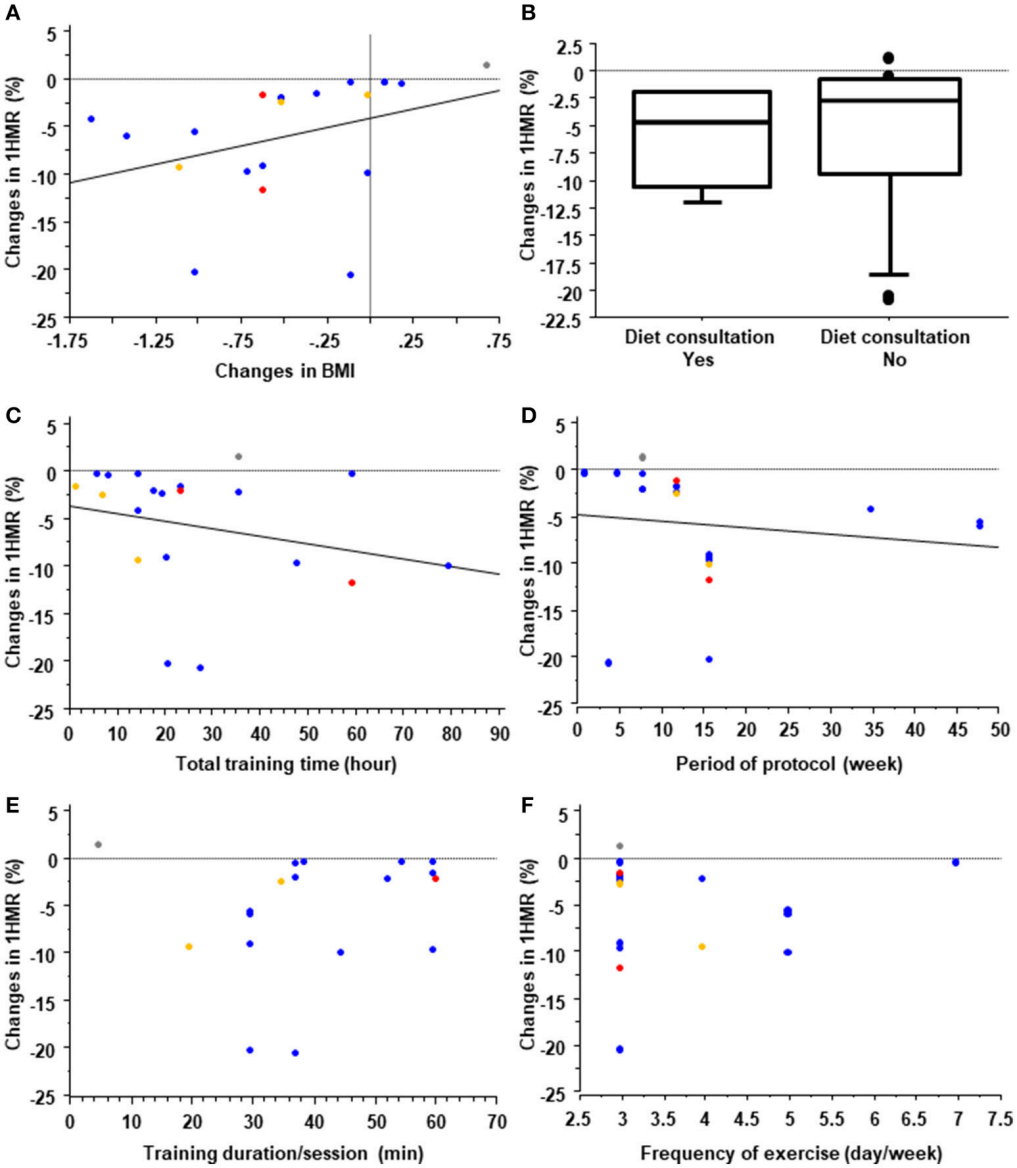
Systematic review of training protocols. Effect of changes in body mass index (BMI) **(A)** and diet consultation **(B)** on liver steatosis measured by ^1^H magnetic resonance (^1^HMR) imaging in exercise training programs for treating patients with nonalcoholic fatty liver disease (NAFLD). In **(A)**, the blue, red, yellow, and gray dots represent training protocols with aerobic, resistance, aerobic plus resistance, and stretching exercises, respectively. Effects of the total training time **(C)**, protocol period **(D)**, training duration/session **(E)**, and frequency of training **(F)** on liver steatosis measured by 1HMR imaging in exercise training programs for subjects with NAFLD. Blue, red and yellow dots represent protocols with aerobic, resistance and combined exercise training, respectively.

Exercise training is routinely recommended for the treatment and management of NAFLD (37, 38). Following the development of imaging modalities to evaluate liver steatosis such as ^1^HMR, conventional B-mode ultrasonography, and controlled attenuation parameters based on transient elastography, liver steatosis has been used as an endpoint of exercise training in many clinical studies. On the basis of findings from the studies of exercise training and other lifestyle modifications, Hannah et al. concluded that a 3% or more body weight reduction with lifestyle modifications ameliorates liver steatosis (39). Indeed, of the 39 exercise training protocols we reviewed, four ineffective ones were found, of which three were without significant body weight reduction and all were without dietary consultation (Supplementary Material 2). In this context, the question is raised of whether the effect of exercise on liver steatosis is independent of nutritional control and body weight reduction in NAFLD. In their systematic review, Hashida et al. suggested that reduction of liver steatosis by aerobic training was observed without a clinically significant weight loss, suggesting that exercise alone might independently reduce hepatic steatosis (40). Of the 39 protocols we reviewed, 22 evaluated changes in liver steatosis (%) using ^1^HMR and assessed their correlations with changes in BMI (Figure 1A). Although our results showed a significant positive correlation between changes in liver fat and changes in BMI (ρ = 0.63, *p* = 0.004), several studies reported an improvement in hepatic steatosis without BMI reduction. Moreover, there was no significant difference in changes in hepatic steatosis between protocols with and without diet consultation (Figure 1B). These findings suggest that exercise *per se* might independently ameliorate hepatic steatosis.

### Clinical question 2. what is the optimal type and dose of exercise for NAFLD therapy?

Aerobic training protocols, resistance training protocols and combined protocols are all effective on ameliorating hepatic steatosis in patients with NAFLD. For aerobic training, walking, jogging with or without a treadmill and ergometer exercise were generally performed. In the protocols of resistance training, major muscles were generally trained using the biceps curl, calf raise, triceps press, chest press, seated hamstrings curl, shoulder press, leg extension, and other exercises. A recent systematic review confirmed that there are no significant differences between aerobic training and resistance training in the extent to which they decrease liver steatosis, as measured by ^1^HMR (40). That review also indicated that the median effective aerobic exercise protocol was 4.8 metabolic equivalents for 40 min/session, 3 times/week for 12 weeks, and the median effective resistance training protocol was 3.5 metabolic equivalents for 45 min/session, 3 times/week for 12 weeks. However, the optimal doses and intensity of exercise training remain unclear. In this context, the EASL-EASD-EASO Clinical Practice Guidelines recommend “moderate exercise” for “150–200 minutes/week” that includes aerobic and resistance exercise (38). In our systematic review, we found no significant differences in the duration of sessions, frequency, protocol period, or total training time between effective and ineffective protocols for liver steatosis (Supplementary Material 3); however, there was a significant negative correlation between changes in liver steatosis measured with ^1^HMR and total training time (Figure 1C; ρ = −0.38, *p* = 0.049) and duration of the exercise protocol (Figure 1D; ρ = −0.59, *p* = 0.007), and no significant correlation between changes in liver steatosis and the duration of each session (Figure 1E; ρ = 0.24, *p* = 0.351) or frequency/week (Figure 1F; ρ = 0.06, *p* = 0.80). This suggests that at least total exercise duration and amount might be important for ameliorating liver steatosis. It is well known that adherence to lifestyle modifications including exercise decreases over time (41). Therefore, keeping the patients motivated for as long as possible and maintaining adherence to protocols is key to the success of exercise therapy for those with NAFLD.

## Mechanisms by which exercise improves NAFLD; a narrative review

Increasing energy expenditure in exercise sessions promotes glucose and lipid metabolism and ameliorates obesity and NAFLD. Based on clinical studies, experimental research has focused on the effect of exercise and training on liver functions, independent of body weight reduction. Numerous studies have demonstrated that exercise and training have a beneficial effect on liver function. In this section, classical and novel exercise training effects on liver function and NAFLD are summarized.

### Classical effects of training on liver metabolism

**Figure 2:**
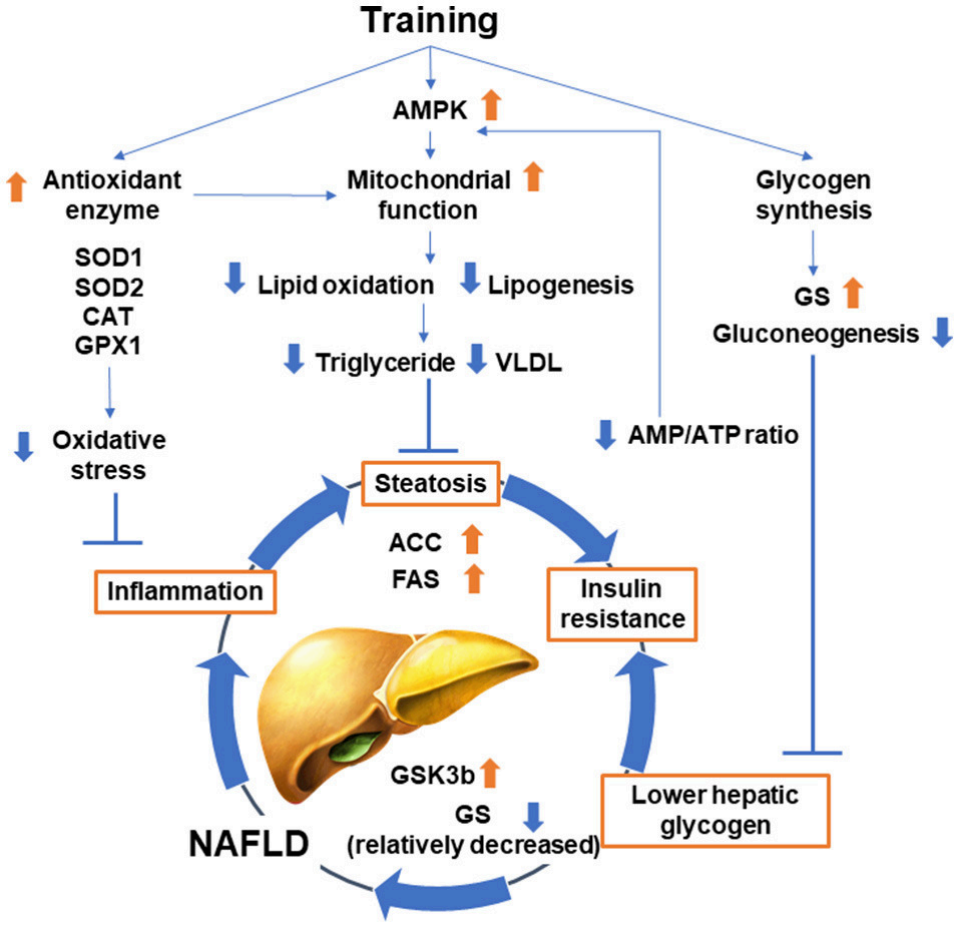
Classical effect of training on subjects with NAFLD.

A number of studies have analyzed the effect of exercise training on liver functions (Figure 2). These began with an analysis of lipid metabolism in the 1970s (42). Thus, training reduced plasma and liver triglycerides in obese Zucker rats and high fat diet-fed rats (42, 43). The Otsuka Long-Evans Tokushima Fatty rat model was well analyzed in terms of its response to exercise training. Decreases in the lipogenic proteins fatty acid synthase (FAS) and acetyl-CoA carboxylase (ACC) with relative increases in the deactivation of ACC by phosphorylation were observed (44–47). Training also increased mitochondrial content markers and oxidation in the liver (44–48), which activates adenosine monophosphate-activated protein kinase (AMPK) and decreases lipogenic processes with a complementary increase in lipid oxidation.

The liver supplies energy substrates to peripheral tissues by glycogen catabolism; therefore, the effect of training on glycogen metabolism has also been studied. Training reduces gluconeogenesis and has a glycogen-sparing effect on the liver to maintain glucose homeostasis during exercise (49, 50). Hepatic glycogen is reduced in subjects with obesity and diabetes by activating hepatic glycogen synthase kinase 3β, which suppresses glycogen synthase (51). Moreover, the increased synthesis of liver glycogen improved the metabolic phenotype of high fat diet-fed mice (52). Taken together, increasing hepatic glycogen might be one of the mechanisms by which training ameliorates hepatic insulin resistance and NAFLD. Increased glycogen contributes to a decrease in the AMP/ATP ratio, which activates AMPK (53, 54). Correlation between glycogen synthesis in skeletal muscle and hepatic *de novo* lipogenesis was also reported. It is well established that exercise increases glycogen synthesis in skeletal muscle, and insulin resistance in skeletal muscle reduces glycogen synthesis because of defects in insulin-stimulated glucose transport activity in skeletal muscle (55, 56). Rabøl et al. demonstrated that a single bout of exercise improved postprandial skeletal muscle glycogen synthesis concomitant with decreased postprandial *de novo* lipogenesis and hepatic triglyceride synthesis in young, lean, insulin-resistant individuals (56). Their finding suggests that improvements in insulin resistance and increased glycogen synthesis in skeletal muscle induced by exercise training or pharmacological therapy can be a therapeutic strategy for patients with NAFLD.

While training decreases hepatic gluconeogenesis, it is well known that the hepatic capacity for gluconeogenesis, as well as the lactate transport capacity and oxidative capacity, are increased by training (57). Training also increases antioxidant enzymes including superoxide dismutase-1 (SOD1) and SOD2, catalase (CAT) and glutathione peroxidase in the liver, and oxidative damage is reduced (58–60). This antioxidant effect is a possible mechanism for the effect of training on NAFLD, which is characterized by hepatic steatosis, inflammation, and oxidative damage (61). In the metabolism of amino acids, training reduced the hepatic catabolism of branched-chain amino acids in rats with streptozotocin-induced diabetes (62).

### Organ crosstalk and novel mechanisms of the effect of training on liver functions

**Figure 3:**
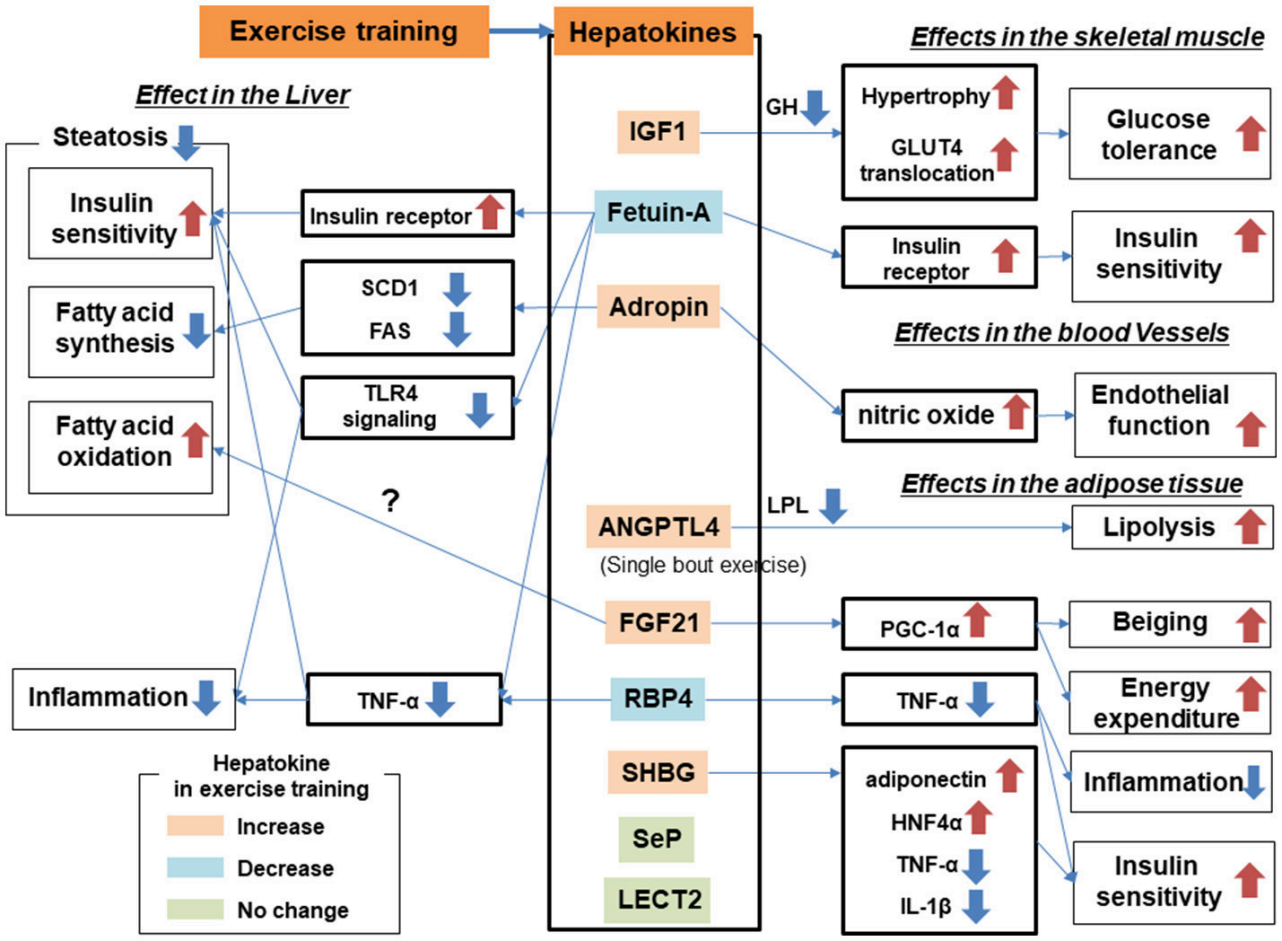
Hepatokines and organ crosstalk in exercise training for subjects with nonalcoholic fatty liver disease (NAFLD).

Training affects multiple organs in addition to skeletal muscle. Many studies have identified organ crosstalk involving the liver, which is a possible mechanism for NAFLD amelioration. In terms of the direction of organ crosstalk involving the liver, the training effect can be categorized as liver to other organs or other organs to liver. Recently, the term “hepatokine” has been proposed to describe the proteins secreted from hepatocytes (63, 64). Because the liver is one of the major endocrine organs, hormonal crosstalk involving growth factors from the liver to other organs has already been studied in the context of the training effect (Table 1, Figure 3). Proteins secreted from adipose tissue known as adipokines and those from skeletal muscle known as myokines are also putative factors in the effect of exercise training on ameliorating NAFLD. Secreted proteins induced by exercise training can be used as a “training biomarker” of NAFLD.

**Table 1:** Hepatokines and exercise training.

**Hepatokine**	**Effect on metabolism and NAFLD**	**Secretion in NAFLD and obesity**	**Changes of secretion or blood concentration by exercise training**			
			**Experimental model**		**Clinical study**	
			**Significance**	**Protocol (ref)**	**Significance**	**Protocols (ref)**
Adropin	Positive	Decreased	–	–	Increase (plasma)	**Ergometer** 55 min, 3 days/weeks, 8 weeks (D42). **Aerobic training** 90 min, 3-5 days/weeks. 12 weeks (D43)
ANGPTL4	Negative/ positive	Decreased	Increase (liver mRNA)	**Treadmill** 60 min, single bout (D55)	Increase (plasma)	**One-legged cycling** 60-180 min, single bout (D 45) **Endurance exercise** 120 min, single bout (D54) **Knee-extensor exercise** 120 min, single bout (D44)
SHBG	Positive	Decreased	–	–	Mostly Increase (serum)	**Walking** 30-45 min, daily, 3 weeks s with diet therapy (D64) **Line dance** 60 min, 3 days/weeks, 16 weeks (D65)
Fetuin A	Negative	Increased	Increase up to normal level (serum)	**Treadmill** 60 min, 5 days/weeks, 16 (D80)	Decrease (serum, plasma)	**Ergometer** 60 min, 5 days/weeks, 12 weeks (D75, D76) **Walking** 60 min, daily, 1 weeks (D77)
FGF21	Positive	Increased	Increase in acute exercise (serum and liver mRNA)	**Treadmill** 30 min, single bout (E12) *Running wheel 8 weeks (E13)	Increase (plasma)	**Treadmill running** 60 min, single bout (D79) **Treadmill running** 30 min, single bout (E12)
Hepassocin	Negative	Increased	–	–	–	–
LECT2	Negative	Increased	–	–	No change (plasma)	**Treadmill running** 60 min, single bout (D79)
RBP4	Negative	Increased	Probably decrease	**Treadmill** 60 min, 5 days/weeks, 10 weeks (D99)	Decrease (serum, plasma)	**Resistance training** 5 days/weeks, 12 weeks (D98) **Stepping training** 60 min, 3 days/weeks, 10 weeks (D100)
Selenoprotein P	Negative	Increased	No change (plasma and liver mRNA)	**Treadmill** 30 min, 6 days/weeks, 1 weeks (F3) **Treadmill** 30 min, 5 days/weeks, 4 weeks (F3)	No change (plasma)	**Treadmill running** 60 min, single bout (D79) **Military training** 360 min, 5 days/weeks, 12 weeks (F2) **Cycling and walking** 30-45 min, 3 days/weeks, 8 weeks (F3)

*non-significant protocol.

### Exercise training-induced protein secretion from liver: hepatokines

Insulin-like growth factor (IGF)-1 is released from the liver in response to hypothalamic hormones. In humans, the serum IGF-1 concentration is lower in patients with diabetes and NAFLD than in healthy subjects (65, 66). Moreover, serum IGF-1 concentrations correlated negatively with the severity of liver fibrosis in patients with NAFLD (67). Zanconato identified that *Igf-1* mRNA expression was enhanced by resistance training in rats (68). In alloxan-induced diabetic rats, serum and hepatic IGF-1 concentrations were reduced compared with those of control rats but recovered to control levels after 8 weeks-of swimming training (69). Training also increased serum IGF-1 concentrations in humans (70). IGF-I stimulates insulin-like actions *in vitro*, including glucose transport, glucose oxidation and translocation of the glucose transporter GLUT-4 to the plasma membrane (71). Skeletal muscle is particularly sensitive to IGF-1 reactions that decrease blood glucose concentrations (65). In addition, a deficiency of IGF-1 *in vivo* results in increased concentrations of growth hormone (GH). These high GH concentrations lead to anti-insulin effects in both liver and adipose tissues, which increase insulin resistance (72). IGF-1 plays an important role in exercise training and increasing IGF-1 levels mediate a lowering of the GH concentration in terms of skeletal muscle growth and repair (73). It mediates protein kinase B activation and concomitantly promotes protein synthesis and inhibits protein degradation (74). It also modulates muscle growth, promoting muscle cell activation, differentiation and hypertrophy (75–77). Moreover, skeletal muscle mass has been linked to the pathogenesis of NAFLD; thus, sarcopenia was an independent risk factor for nonalcoholic steatohepatitis (NASH; nonalcoholic steatohepatitis) and NAFLD with severe fibrosis (78, 79). Taken together, increased or sustained IGF-1 concentrations in the blood and liver are putative factors in the effect of exercise training on ameliorating NAFLD.

Adropin consists of 76 amino acids and has been linked to metabolic homeostasis, cardiovascular function and endothelial cell function (80, 81). It is expressed in multiple tissues, including the brain, heart, kidney, liver, pancreas, skeletal muscle, and small intestine (82, 83). Kumar et al. suggested that adropin is an hepatokine that is decreased in diet-induced obese mice and showed that transgenic overexpression or systemic adropin treatment attenuated steatosis by suppressing the expression of *Fas* and the gene for Stearoyl-CoA desaturase-1 (*Scd1*) (80). Aerobic training increased serum adropin levels in humans, and this was associated with reduced arterial stiffness (84) and improvements in endothelial function (85).

Angiopoietin-like protein 4 (ANGPTL4) is secreted from multiple tissues including the liver (86) and is considered to be an exercise-induced hepatokine (87). ANGPTL4 has been associated with lipid homeostasis (86, 88), but its effect on glucose metabolism remains equivocal (89, 90). ANGPTL4 was found to stimulate lipolysis (91) and to inhibit the clearance of triglycerides from plasma by inhibiting lipoprotein lipase (92, 93). ANGPTL4 overexpression in either normal chow diet-fed mice or high fat diet-fed mice reduced the weight of adipose tissue but increased liver steatosis and elevated plasma triglycerides, free fatty acids, glycerol, total cholesterol and high-density lipoprotein cholesterol (89, 90). Therefore, the effect of ANGPTL4 on NAFLD can be explained as being both positive and negative: increasing liver steatosis and decreasing adiposity. Plasma ANGPTL4 concentration was increased by a single bout of acute exercise but not by chronic exercise or training in humans (87, 88, 94). Increased *Angptl4* mRNA expression in liver was also recognized in mice after treadmill exercise (95). During exercise, ANGPTL4 levels are positively regulated by free fatty acids, glucagon and cAMP, and negatively regulated by AMPK (87, 88). ANGPTL4 is also related to the microbiome; thus, conventionalization of germ-free mice suppressed ANGPTL4 expression in gut epithelial cells (96). Further research is required to elucidate the link between ANGPTL4 and the microbiome in cases of NAFLD.

Circulating sex hormone-binding globulin (SHBG) secreted from the liver regulates the biological action and signaling of sex hormones (97). The relationship between these hormones and glucose homeostasis is complicated. For example, testosterone levels correlate positively with insulin resistance, glucose intolerance and an increased risk of type 2 diabetes in women, whereas the opposite appears to be true in men. Conversely, high estradiol levels are associated with elevated insulin resistance and increased risk of type 2 diabetes in both genders (98, 99). The relationship between SHBG and glucose metabolism is more consistent than that between the sex hormones and glucose metabolism across the genders. Circulating SHBG concentrations correlate positively with insulin sensitivity in humans, suggesting that circulating SHBG might prevent the development of type 2 diabetes (100). In a cross-sectional study that measured plasma SHBG in 233 dysmetabolic men, there was a significant correlation between plasma SHBG concentration and intrahepatic fat measured by ultrasonography (101). In addition, circulating SHBG increased with lifestyle modifications including diet control and aerobic exercise, and the response to increasing SHBG correlated more strongly with decreasing liver steatosis than with visceral adiposity (100). According to *in vitro* experiments, adiponectin positively regulates SHBG production in hepatocytes through the transcription factor hepatocyte nuclear factor 4α (102). SHBG also suppresses proinflammatory cytokines including interleukin (IL)-1β (103) and tumor necrosis factor-alpha (TNF-α) (104), reactions that are mediated by hepatocyte nuclear factor 4α. Changes in SHBG after training have been analyzed, with some reporting an increase in circulating SHBG concentrations (105, 106). Daily walking for 3 weeks combined with dietary therapy increased serum SHBG by 38% in obese men (105). Aerobic exercise training for 16 weeks increased serum SHBG by 6% in obese postmenopausal women (106). However, a single bout of running exercise for 45 min in healthy, physically active men showed only a nonsignificant tendency for increased serum SHBG (107).

Alpha-2-HS-glycoprotein, also known as fetuin-A, shows diverse functions including osteogenesis and bone resorption, and regulation of the insulin and hepatocyte growth factor receptors and responses to systemic inflammation (64, 108). Fetuin-A is predominantly secreted by the liver (109) and inhibits the insulin receptor tyrosine kinase in liver and skeletal muscle (110, 111). Mice with deletion of the *Ahsg* gene, encoding fetuin-A, showed improved insulin signaling (112). Fetuin-A is also an adaptor protein for saturated fatty acids, allowing them to activate Toll-like receptor 4 and increase insulin resistance (113). A clinical study confirmed a positive correlation between circulating fetuin-A concentrations and insulin resistance (114). Exercise training including aerobic exercise using an ergometer or walking generally reduces (115–117) or tends to reduce (118, 119) circulating fetuin-A in subjects with type 2 diabetes and NAFLD. Improvements in hepatic insulin resistance correlated with decreasing levels of blood fetuin-A (116). Sedentary and cholesterol diet-fed mice with low-density lipoprotein receptor deficiency showed a lower serum fetuin-A concentration than sedentary control mice fed a normal chow diet, but treadmill running (60 min/day, 5 days/week) for 16 weeks negated the dietary effect of cholesterol by increasing the serum fetuin-A concentration to the level of control mice (120). According to those findings, training generally reduces the increased circulating fetuin-A in obesity but increases fetuin-A in the case of low-density lipoprotein receptor deficiency. Fetuin-B, also considered to be a hepatokine, has been shown to impair glucose tolerance and is associated with hepatic steatosis in mice (121). Further research is required to elucidate the effect of exercise training on the regulation of fetuin-B.

Hepassocin is important for the regeneration and proliferation of hepatocytes, acting through extracellular signal-regulated kinase 1/2 (122–124). It appears to be a hepatokine (125) and is related to glucose intolerance and insulin resistance. Hepassocin levels are increased in human subjects with prediabetes, type 2 diabetes, and NAFLD (126, 127). Administration of recombinant hepassocin increased NAFLD activity including steatosis and induced insulin resistance in both liver and skeletal muscle tissues (126). To date, there have been no reports analyzing the effect of exercise training on hepassocin expression or secretion.

Leukocyte cell-derived chemotaxin 2 (LECT2) was originally identified as a neutrophil chemotactic protein (127) and is considered to be a hepatokine (128, 129). It impairs insulin signaling and increased c-jun *N*-terminal kinase signaling, suggesting that LECT2 has a pro-inflammatory role (130). Indeed, deletion of *Lect2* in mice improved high fat diet-induced insulin resistance with decreased c-jun N-terminal kinase signaling in skeletal muscle (130). In humans, serum LECT2 concentrations correlated positively with insulin resistance (130). Changes in circulating LECT2 or secretion of LECT2 from liver during exercise training have not been well-studied. A single bout of exercise on a moderate-intensity treadmill for 1 h failed to increase the serum LECT2 concentration in humans (119).

Although serum fibroblast growth factor 21 (FGF21) is predominantly secreted from the liver, it has also been found in the pancreas, testis, duodenum and adipose tissue. Administration of FGF21 improved the metabolic phenotype and reduced hepatic triglyceride levels in high fat diet-fed mice, diabetic monkeys, and humans with diabetes (131, 132). Hepatocytes are a main source of FGF21; thus, FGF21 is considered to be a hepatokine. Fletcher et al. tested the effect of FGF21 on exercise-induced hepatic mitochondrial adaptations in FGF21 knockout mice (133). FGF21 gene knockout mice showed 30–50% lower hepatic mitochondrial complete palmitate oxidation, β-hydroxyacyl-CoA dehydrogenase activity, and nuclear content of peroxisome proliferator-activated receptor gamma coactivator 1-alpha (PGC-1α) in the sedentary condition; however, the effect of exercise on these markers was minimal. Although the direct effect of FGF21 on liver is still controversial because of the lack of an FGF receptor 1 in hepatocytes, NAFLD might be alleviated through the effect of training on adipose tissues: namely, the development of brown fat-like cells in white adipose tissue (the “beiging” phenomenon) and increased levels of adiponectin (134, 135). A single bout of exercise increased circulating FGF21 levels in mice and humans and FGF21 mRNA expression in the liver of mice (119, 136), whereas wheel running failed to increase FGF21 mRNA and protein expression in the livers of mice (137). This suggests that acute rather than chronic exercise or training contributes to increasing hepatic FGF21 production.

Retinol-binding protein 4 (RBP4) is secreted from hepatocytes and adipocytes and is considered to be both a hepatokine and an adipokine (138). It was originally identified as a transport protein for vitamin A (139), and its expression is linked to obesity, metabolic syndrome, and insulin resistance. Serum RBP4 concentration correlated positively with the magnitude of insulin resistance in subjects with obesity or diabetes (140). Elevated serum RBP4 levels were also associated with components of the metabolic syndrome (140). Transgenic overexpression of human RBP4 or injection of recombinant RBP4 in normal mice caused insulin resistance (138), whereas deletion of RBP4 enhanced insulin sensitivity (138, 141). Improvement of metabolic syndrome with diet therapy and bariatric surgery decreased serum RBP4 concentrations (142, 143). Exercise training also reduced circulating RBP4 levels and resistance training decreased circulating RBP4 in people with type 2 diabetes (144). Training with aerobic exercise for 10 weeks also decreased the circulating RBP4 levels in healthy women (145), whereas a single bout of resistance exercise failed to decrease the RPB4 concentration (146). In spontaneously hypertensive rats with insulin resistance, treadmill running reduced circulating RBP4 concentrations (147). Circulating RBP4 decreased in streptozotocin-induced diabetic rats (148). Interestingly, RBP4 mRNA expression decreased in visceral fat tissue but not in the liver, suggesting that adipocytes are predominant in the response of circulating RBP4 levels to training.

Selenoprotein P (SeP) is a liver-derived secretory protein, and a significant positive correlation in humans between SeP mRNA expression and insulin resistance was identified using serial analysis of gene expression and DNA chip methods (149). SeP-deficient mice showed more endurance capacity after training through upregulation of reactive oxygen species and AMPK (150); however, there was no change in SeP secretion with exercise training in rodents (150) or humans (119, 151), suggesting that inhibition of SeP is an exercise-enhancer rather than an exercise mimicker.

### Effects of training through adipokines and myokines on the liver and NAFLD

Regular physical activity and exercise training have long been known to cause adaptations to white adipose tissue, including decreases in cell size and lipid content and increases in mitochondrial proteins (152). Exercise training also alters adipokine secretion. According to a recent systematic review including 1774 obese subjects, exercise training significantly reduced serum leptin and increased adiponectin concentrations (153). Leptin regulates appetite through an afferent signal, and acute intravenous or intracerebroventricular administrations of leptin increased glucose turnover and glucose uptake independently of the blood insulin and glucose levels (154). Leptin treatment improved insulin resistance and diabetes in mice with congenital lipodystrophy (155). In general, serum leptin levels correlated positively with adiposity and hyperleptinemia and leptin resistance were observed in obese subjects (156, 157). In the liver, leptin directly promotes fibrogenesis. Leptin induced transforming growth factor β (TGF-β) in hepatic stellate cells through indirect effects on Küpffer cells in an animal model (158). Therefore, reducing circulating leptin levels by exercise training might contribute to ameliorating liver fibrosis in subjects with NAFLD. On the other hand, no association between circulating leptin levels and the severity of liver fibrosis has been confirmed in human (159). Adiponectin is secreted from adipose tissues and is abundant in serum (160). Adiponectin negatively correlates with serum triglyceride and with apolipoprotein B (ApoB) levels, which is a triglyceride-rich very-low-density lipoprotein (VLDL) (161, 162). In hepatocytes, adiponectin reduced triglyceride and ApoB levels and served to reduce VLDL secretion from the liver (163). Numerous studies have revealed the beneficial effect of adiponectin on the pathogenesis of NAFLD (164). Adiponectin administration suppressed the expression of sterol regulatory element-binding protein (SREBP) 1c in the liver of leptin-receptor deficient (db/db) mice as well as in cultured hepatocytes (165). Choline and l-amino acid-deficient diet fed-mice showed more severe hepatic steatosis in adiponectin-deficient mice than in wild type mice (166). In db/db mice and high fat diet-fed mice, reduced adiponectin signaling genes and protein expression including adiponectin receptor levels were linked with the severe hepatic phenotype of NASH, reduced mitochondrial biogenesis markers and reduced AMPK signaling (167). Peroxisome proliferator-activated receptor alpha (PPARα) is a key regulator of lipid metabolism and associates with fatty acid oxidation in the liver. In human subjects with NASH, hepatic expression of the gene encoding PPARα was correlated positively with serum adiponectin levels (168). Serum adiponectin levels were negatively correlated with hepatic steatosis in such subjects (169). Adiponectin has demonstrated beneficial effects against hepatic inflammation and fibrosis. In several mouse models of immune-mediated hepatitis, adiponectin reduced TNF levels and induced IL-10 release from Küpffer cells (170). Lower nuclear factor kappa B (NFκB) levels were also reported (171, 172). Adiponectin receptor 2 (AdipoR2)-deficient mice fed a methionine-choline deficient diet showed higher levels of steatosis, inflammation and fibrosis (173). Moreover, overexpression of AdipoR2 inhibited TGF-β signaling and stimulation of PPARα activity (173). Adiponectin reduced the proliferation of human stellate cells and lowered the levels of alpha smooth muscle actin induced in activated hepatic stellate cells (174). Adiponectin also inhibited leptin-induced STAT3 phosphorylation in activated hepatic stellate cells and leptin-mediated upregulation of tissue inhibitor of metalloproteinase 1 (TIMP-1) release both *in vitro* and *in vivo* (175). These studies suggest that adiponectin ameliorates hepatic steatosis, inflammation and fibrosis in NAFLD through multiple mechanisms and increased adiponectin levels by exercise training is one potential explanation for the benefit of exercise training on NAFLD.

Perilipin 5 (PLIN5) is a lipid droplet-associated protein that is highly expressed in oxidative tissue. In high fat diet-fed mice trained on a treadmill, mice with muscle-specific PLIN5 overexpression showed decreased liver fat and mRNA expression of genes encoding proinflammatory cytokines (176). In these mice, the increase in serum FGF21 was double that of the control mice, suggesting that increased PLIN5 expression might mediate an increase in the levels of circulating FGF21 after training.

IL-6 is released from contracting muscle, and was first identified as a myokine (177, 178). In skeletal muscle, IL-6 increases glucose uptake and fatty acid oxidation through activation of AMPK and/or phosphatidylinositol-3-kinase (PI3-kinase) pathways (179). Circulating IL-6 released from skeletal muscle directly affects whole body metabolism in distant organs. In adipose tissues, IL-6 induces lipolysis and increases fatty acid oxidation through activation of AMPK (180). IL-6 also increases the proliferation of pancreatic β cells and increases glucose-stimulated insulin secretion from them (181, 182). In the liver, muscle-derived IL-6 enhances hepatic glucose production during exercise (183) and has been reported to upregulate the expressions of gluconeogenic genes directly leading to increased hepatic glucose production (183, 184). These action of IL-6 in the liver might contribute to maintain glucose homeostasis during exercise. Indeed, circulating IL-6 levels negatively correlates with those of plasma glucose during exercise in humans (185), suggesting that IL-6 might be a sensor of carbohydrate availability (186). L-6 infusion reduced hepatic steatosis and ischemia/reperfusion injury and promoted the proliferation of hepatocytes in rodent models (187–190). As well as skeletal muscle and adipose tissue, these effects in the liver were linked with an increase in mitochondrial fatty acid oxidation. IL-6 also affected PPARα levels in the liver (188), mediated the levels of fatty acid binding protein and positively regulated PPARα production in the liver (191). PPARα was shown to upregulate the expression of genes including those involved in fatty acid transport and mitochondrial fatty acid oxidation (192). These experimental studies suggest that IL-6 might be involved in the way exercise training alleviates NAFLD. In addition, findings from IL-6-deficient mice, which develop mature-onset obesity, demonstrated a suppressive effect of IL-6 on the development of obesity (191). On the other hand, it is well known that IL-6 is an inflammatory cytokine and serum IL-6 concentrations are generally increased in subjects showing obesity, diabetes and NAFLD (193, 194). TNF-α upregulates obesity-induced IL-6 production and causes hepatic inflammation through activation of extracellular signal-regulated kinase (ERK) and signal transducers and activator of transcription 3 (Stat3) signaling (195). IL-6-deficient mice gained body weight slower than wild type mice under high fat diet-fed-conditions (196). Moreover, IL-6-deficient mice showed less severe steatosis and inflammation in the liver (195). In a clinical study including subjects with NASH, IL-6 was decreased significantly in those subjects who received either aerobic exercise training or resistance exercise training (197). Taken together, there are discrepancies among studies in terms of the effects of IL-6 on obesity and NAFLD, and further research is required to clarify the effects of IL-6 on NAFLD as a myokine and as an inflammatory cytokine.

Irisin is a 112 amino acid proteolytically cleaved form of fibronectin type III domain-containing protein 5 that has been identified as a training-induced secretion factor (135). Irisin is secreted from muscles during or after exercise and induces beiging of white adipose tissue by activating PGC1α, resulting in an improvement in glucose and lipid metabolism in multiple organs (133, 198). The effect of irisin on liver has also been investigated. Recombinant irisin protein significantly inhibited the increase in the palmitic acid-induced lipogenic markers ACC and FAS and prevented palmitic acid-induced lipid accumulation in primary hepatocytes (199). The researchers also identified an anti-inflammatory effect of irisin with reductions in inflammatory mediators including TNF-α, IL-6 and NF-κB, which might be mediated by protein arginine methyltransferase 3, an enzyme actively participating in the hepatic lipogenesis pathway. Serum irisin concentrations were increased in human subjects with NAFLD (200), and this was considered to be a protective compensatory response. Irisin also acts against oxidative stress and serum irisin concentration correlates with hepatic and muscle malondialdehyde levels (201, 202). As for its anti-inflammatory effect, the antioxidative effect of irisin mediates the inhibition of protein arginine methyltransferase 3 (199).

### Other mechanisms of the effects of exercise training on the liver

MicroRNAs (miRNAs) are small untranslated RNA transcripts frequently expressed under the control of nuclear receptors. They are involved in multiple cellular pathways including metabolism. The association between exercise training and miRNAs has been studied. Thus, a comparative analysis of livers from mice subjected to exercise training showed significant changes in miRNAs (203). It was reported that miR-33 positively regulated hepatic fatty acid oxidation and insulin signaling and reduces lipogenesis (204). In high fat diet-fed mice, the expression of hepatic miR-33 was decreased significantly, whereas aerobic exercise on a treadmill for 10 weeks increased miR-33 expression to the level of the normal chow-fed control mice (205). Another miRNA array study on mice showed that increased levels of miR-212 in high fat diet-fed mice was reduced by treadmill running for 16 weeks (203). In that study, a negative correlation between miR-212 and FGF21 levels was also demonstrated in HepG2 cells, suggesting that decreased miR-212 might underlie the effect of exercise training on reducing lipogenesis through increasing FGF21 production (206).

Numerous clinical and experimental studies have indicated a strong correlation between the microbiome and the pathogenesis of NAFLD (207, 208). Lifestyle disturbances including excess “Western-style” diet consumption and diet-induced obesity cause severe microbial dysbiosis and have a direct impact on hepatic metabolism (209). Lipopolysaccharides produced by the Negativicute and Halanaerobiale bacteria, which belong to the Phylum Firmicutes, are associated with the progression of NASH, including liver inflammation and fibrosis (210). Intestinal permeability is involved in the pathogenesis of NAFLD and affects the microbiome (211). Increased intestinal permeability results in increased inflammation-based and bacterial metabolite-driven pathways (212, 213). Lifestyle modifications can affect the microbiome. Indeed, numerous studies have demonstrated that training and physical activity changes the microbiome (214, 215). Both treadmill running and voluntary wheel running increased microbiome diversity in mice, and this effect was also observed in high fat diet-fed mice (216, 217). However, the effect of exercise training on the ratio of Firmicutes to Bacteroidetes, which is generally considered to increase in cases of obesity and diabetes (209, 218), is inconsistent in the literature because of differences in training protocols and sampling locations between studies (214–217). *Bifidobacterium* is a known regulator of intestinal permeability (219, 220) and several studies have reported an increase in *Bifidobacterium* with exercise training (218, 221), suggesting that exercise improves gut barrier function. This suggests that alteration of the microbiome is involved in the effect of exercise training on ameliorating NAFLD, and further research is warranted.

## Future direction of exercise training treatment for alleviating NAFLD

According to recent studies and consensus, liver fibrosis is the most significant factor for determining the prognosis of NAFLD, independent of age and concomitant disease including diabetes (222, 223). Therefore, it is important to identify whether exercise training ameliorates or prevents liver fibrosis and improves the prognosis of subjects with NAFLD. Moreover, it is necessary to investigate the molecular pathways involved in the exercise training effect on the pathogenesis of liver fibrosis. To date, few studies have evaluated liver fibrosis in liver specimens (9, 224). In this context, the development of a noninvasive method to evaluate liver fibrosis in subjects with NASH including magnetic resonance imaging and transient elastography will contribute to further clinical trials targeting liver fibrosis with exercise training (225, 226). In experimental research, comprehensive analyses including gene microarrays, next generation sequencing and metabolomics, developed in the 2000s, have indicated possible molecular mechanisms by which NAFLD might be ameliorated in rodent models and humans (227–230). These technologies are expected to reveal the molecular mechanisms and contribute to translational research on exercise training in subjects with NAFLD. Another aspect of investigation into the effect of exercise training on NAFLD is the potential development of a therapeutic agent as a “training mimicker.” It is well known that maintaining adherence to lifestyle modifications including exercise training and dietary therapy is difficult (41). Moreover, concomitant disease and complications of obesity including diabetes, cardiovascular disease and inactivity linked with orthopedic diseases and aging frequently disrupt exercise training for subjects with NAFLD. Training mimickers would provide these patients with the benefits of exercise training.

## Conclusion

To conclude, exercise training is a robust treatment for subjects with NAFLD. There are multiple mechanisms by which this acts on the liver, including organ crosstalk. Although further clinical research is needed to evaluate the effect of exercise training on liver fibrosis and prognosis for patients with NAFLD, it is important to increase physical activity and promote lifestyle modification for the management of this disorder.

## Author contributions

HT generated the manuscript including the main document, tables and figures. KK initially designed the contents of the sections and supervised writing of the manuscript. KT and HT reviewed and analyzed the literature for this systematic review. YE edited the manuscript and supervised the figure design. KA organized the data and wrote the manuscript as a corresponding author.

### Conflict of interest statement

The authors declare that the research was conducted in the absence of any commercial or financial relationships that could be construed as a potential conflict of interest.

## References

[1] Younossi ZM Koenig AB Abdelatif D Fazel Y Henry L Wymer M. Global epidemiology of nonalcoholic fatty liver disease-Meta-analytic assessment of prevalence, incidence, and outcomes. Hepatology (2016) 64:73–84. 10.1002/hep.28431 26707365

[2] Ekelund U Steene-Johannessen J Brown WJ Fagerland MW Owen N Powell KE. Does physical activity attenuate, or even eliminate, the detrimental association of sitting time with mortality? A harmonised meta-analysis of data from more than 1 million men and women. Lancet (2016) 388:1302–10. 10.1016/S0140-6736(16)30370-1 27475271

[3] Colberg SR Sigal RJ Yardley JE Riddell MC Dunstan DW Dempsey PC. Physical activity/exercise and diabetes: a position statement of the American Diabetes Association. Diabetes Care (2016) 39:2065–79. 10.2337/dc16-1728 27926890 PMC6908414

[4] Kerr J Anderson C Lippman SM. Physical activity, sedentary behaviour, diet, and cancer: an update and emerging new evidence. Lancet Oncol. (2017) 18:e457–71. 10.1016/S1470-2045(17)30411-4 28759385 PMC10441558

[5] Kivimäki M Kuosma E Ferrie JE Luukkonen R Nyberg ST Alfredsson L. Overweight, obesity, and risk of cardiometabolic multimorbidity: pooled analysis of individual-level data for 120813 adults from 16 cohort studies from the USA and Europe. Lancet Public Health (2017) 2:e277–85. 10.1016/S2468-2667(17)30074-9 28626830 PMC5463032

[6] Patnode CD Evans CV Senger CA Redmond N Lin JS. Behavioral counseling to promote a healthful diet and physical activity for cardiovascular disease prevention in adults without known cardiovascular disease risk factors: updated evidence report and systematic review for the US preventive services task force. JAMA (2017) 318:175–93. 10.1001/jama.2017.3303 28697259 PMC13292382

[7] Ryu S Chang Y Jung HS Yun KE Kwon MJ Choi Y. Relationship of sitting time and physical activity with non-alcoholic fatty liver disease. J Hepatol. (2015) 63:1229–37. 10.1016/j.jhep.2015.07.010 26385766

[8] Sung KC Ryu S Lee JY Kim JY Wild SH Byrne CD. Effect of exercise on the development of new fatty liver and the resolution of existing fatty liver. J Hepatol. (2016) 65:791–7. 10.1016/j.jhep.2016.05.026 27255583

[9] Ueno T Sugawara H Sujaku K Hashimoto O Tsuji R Tamaki S. Therapeutic effects of restricted diet and exercise in obese patients with fatty liver. J Hepatol. (1997) 27:103–7. 10.1016/S0168-8278(97)80287-5 9252081

[10] Chen SM Liu CY Li SR Huang HT Tsai CY Jou HJ. Effects of therapeutic lifestyle program on ultrasound-diagnosed nonalcoholic fatty liver disease. J Chin Med Assoc. (2008) 71:551–88. 10.1016/S1726-4901(08)70168-0 19015052

[11] Kantartzis K Thamer C Peter A Machann J Schick F Schraml C. High cardiorespiratory fitness is an independent predictor of the reduction in liver fat during a lifestyle intervention in non-alcoholic fatty liver disease. Gut (2009) 58:1281–8. 10.1136/gut.2008.151977 19074179

[12] Johnson NA Sachinwalla T Walton DW Smith K Armstrong A Thompson MW. Aerobic exercise training reduces hepatic and visceral lipids in obese individuals without weight loss. Hepatology (2009) 50:1105–12. 10.1002/hep.23129 19637289

[13] Vilar Gomez E Rodriguez De Miranda A Gra Oramas B Arus Soler E Llanio Navarro R Calzadilla Bertot L. Clinical trial: a nutritional supplement Viusid, in combination with diet and exercise, in patients with nonalcoholic fatty liver disease. Aliment Pharmacol Ther. (2009) 30:999–1009. 10.1111/j.1365-2036.2009.04122.x 19691668

[14] Slentz CA Bateman LA Willis LH Shields AT Tanner CJ Piner LW. Effects of aerobic vs. resistance training on visceral and liver fat stores, liver enzymes, and insulin resistance by HOMA in overweight adults from STRRIDE AT/RT. Am J Physiol Endocrinol Metab. (2011) 301:E1033–9. 10.1152/ajpendo.00291.2011 21846904 PMC3214001

[15] Sullivan S Kirk EP Mittendorfer B Patterson BW Klein S. Randomized trial of exercise effect on intrahepatic triglyceride content and lipid kinetics in nonalcoholic fatty liver disease. Hepatology (2012) 55:1738–45. 10.1002/hep.25548 22213436 PMC3337888

[16] Bhat G Baba CS Pandey A Kumari N Choudhuri G Life style modification improves insulin resistance and liver histology in patients with nonalcoholic fatty liver disease. World J Hepatol. (2012) 4:209–17. 10.4254/wjh.v4.i7.209 22855696 PMC3409355

[17] Bacchi E Negri C Targher G Faccioli N Lanza M Zoppini G. Both resistance training and aerobic training reduce hepatic fat content in type 2 diabetic subjects with nonalcoholic fatty liver disease (the RAED2 Randomized Trial). Hepatology (2013) 58:1287–95. 10.1002/hep.26393 23504926

[18] Haus JM Solomon TP Kelly KR Fealy CE Kullman EL Scelsi AR. Improved hepatic lipid composition following short-term exercise in nonalcoholic fatty liver disease. J Clin Endocrinol Metab. (2013) 98: E1181. 10.1210/jc.2013-1229 23616151 PMC3701282

[19] Khaoshbaten M Gholami N Sokhtehzari S Monazami AH Nejad MR. The effect of an aerobic exercise on serum level of liver enzymes and liver echogenicity in patients with non alcoholic fatty liver disease. Gastroenterol Hepatol Bed Bench (2013) 6(Suppl. 1):S112–6. 10.22037/ghfbb.v6i0.482 24834280 PMC4017540

[20] Yoshimura E Kumahara H Tobina T Matsuda T Ayabe M Kiyonaga A. Lifestyle intervention involving calorie restriction with or without aerobic exercise training improves liver fat in adults with visceral adiposity. J Obes. (2014) 2014:197216. 10.1155/2014/197216 24864199 PMC4016916

[21] Oh S Shida T Yamagishi K Tanaka K So R Tsujimoto T. Moderate to vigorous physical activity volume is an important factor for managing nonalcoholic fatty liver disease: a retrospective study. Hepatology (2015) 61:1205–15. 10.1002/hep.27544 25271091

[22] Keating SE Hackett DA Parker HM O'Connor HT Gerofi JA Sainsbury A. Effect of aerobic exercise training dose on liver fat and visceral adiposity. J Hepatol. (2015) 63:174–82. 10.1016/j.jhep.2015.02.022 25863524

[23] Hallsworth K Thoma C Hollingsworth KG Cassidy S Anstee QM Day CP. Modified high-intensity interval training reduces liver fat and improves cardiac function in non-alcoholic fatty liver disease: a randomized controlled trial. Clin Sci. (2015) 129:1097–105. 10.1042/CS20150308 26265792

[24] Shamsoddini A Sobhani V Ghamar Chehreh ME Alavian SM Zaree A. Effect of aerobic and resistance exercise training on liver enzymes and hepatic fat in Iranian men with nonalcoholic fatty liver disease. Hepat Mon. (2015) 15:e31434. 10.5812/hepatmon.31434 26587039 PMC4644631

[25] Lee S Bacha F Hannon T Kuk JL Boesch C Arslanian S Effects of aerobic vs. resistance exercise without caloric restriction on abdominal fat, intrahepatic lipid, and insulin sensitivity in obese adolescent boys: a randomized, controlled trial. Diabetes (2012) 61:2787–95. 10.2337/db12-0214 22751691 PMC3478522

[26] Fealy CE Haus JM Solomon TP Pagadala M Flask CA McCullough AJ. Short-term exercise reduces markers of hepatocyte apoptosis in nonalcoholic fatty liver disease. J Appl Physiol. (2012) 113:1–6. 10.1152/japplphysiol.00127.2012 22582214 PMC3404833

[27] Cassidy S Thoma C Hallsworth K Parikh J Hollingsworth KG Taylor R. High intensity intermittent exercise improves cardiac structure and function and reduces liver fat in patients with type 2 diabetes: a randomised controlled trial. Diabetologia (2016) 59:56–66. 10.1007/s00125-015-3741-2 26350611 PMC4670457

[28] Cuthbertson DJ Shojaee-Moradie F Sprung VS Jones H Pugh CJ Richardson P. Dissociation between exercise-induced reduction in liver fat and changes in hepatic and peripheral glucose homoeostasis in obese patients with non-alcoholic fatty liver disease. Clin Sci. (2016) 130:93–104. 10.1042/CS20150447 26424731

[29] Houghton D Thoma C Hallsworth K Cassidy S Hardy T Burt AD. Exercise reduces liver lipids and visceral adiposity in patients with nonalcoholic steatohepatitis in a randomized controlled trial. Clin Gastroenterol Hepatol. (2017) 15:96–102.e3. 10.1016/j.cgh.2016.07.031 27521509 PMC5196006

[30] Rezende RE Duarte SM Stefano JT Roschel H Gualano B de Sá Pinto AL. Randomized clinical trial: benefits of aerobic physical activity for 24 weeks in postmenopausal women with nonalcoholic fatty liver disease. Menopause (2016) 23:876–83. 10.1097/GME.0000000000000647 27458060

[31] Shojaee-Moradie F Cuthbertson DJ Barrett M Jackson NC Herring R Thomas EL Exercise training reduces liver fat and increases rates of VLDL clearance but not VLDL production in NAFLD. J Clin Endocrinol Metab. (2016) 101:4219–28. 10.1210/jc.2016-2353 27583475

[32] Zhang HJ He J Pan LL Ma ZM Han CK Chen CS. Effects of moderate and vigorous exercise on nonalcoholic fatty liver disease: a randomized clinical trial. JAMA Intern Med. (2016) 176:1074–82. 10.1001/jamainternmed.2016.3202 27379904

[33] Pugh CJ Spring VS Kemp GJ Richardson P Shojaee-Moradie F Umpleby AM. Exercise training reverses endothelial dysfunction in nonalcoholic fatty liver disease. Am J Physiol Heart Circ Physiol. (2014) 307:H1298–306. 10.1152/ajpheart.00306.2014 25193471

[34] Taniguchi H Tanisawa K Sun X Kubo T Higuchi M. Endurance exercise reduces hepatic fat content and serum fibroblast growth factor 21 levels in elderly men. J Clin Endocrinol Metab. (2016) 101:191–8. 10.1210/jc.2015-3308 26562755

[35] Takahashi A Abe K Usami K Imaizumi H Hayashi M Okai K. Simple resistance exercise helps patients with non-alcoholic fatty liver disease. Int J Sports Med. (2015) 36:848–52. 10.1055/s-0035-1549853 26090879

[36] Zelber-Sagi S Buch A Yeshua H Vaisman N Webb M Harari G. Effect of resistance training on non-alcoholic fatty-liver disease a randomized-clinical trial. World J Gastroenterol. (2014) 20:4382–92. 10.3748/wjg.v20.i15.4382 24764677 PMC3989975

[37] Chalasani N Younossi Z Lavine JE Charlton M Cusi K Rinella M. The diagnosis and management of nonalcoholic fatty liver disease: Practice guidance from the American Association for the Study of Liver Diseases. Hepatology (2018) 67:328–57. 10.1002/hep.29367 28714183

[38] European Association for the Study of the Liver (EASL); European Association for the Study of Diabetes (EASD); European Association for the Study of Obesity (EASO) EASL-EASD-EASO Clinical Practice Guidelines for the management of non-alcoholic fatty liver disease. J Hepatol. (2016) 64:1388–402. 10.1016/j.jhep.2015.11.004 27062661

[39] Hannah WN Jr Harrison SA. Effect of Weight loss, diet, exercise, and bariatric surgery on nonalcoholic fatty liver disease. Clin Liver Dis. (2016) 20:339–50. 10.1016/j.cld.2015.10.008 27063273

[40] Hashida R Kawaguchi T Bekki M Omoto M Matsuse H Nago T. Aerobic vs. resistance exercise in non-alcoholic fatty liver disease: a systematic review. J Hepatol. (2017) 66:142–52. 10.1016/j.jhep.2016.08.023 27639843

[41] Oza N Eguchi Y Mizuta T Ishibashi E Kitajima Y Horie H. A pilot trial of body weight reduction for nonalcoholic fatty liver disease with a home-based lifestyle modification intervention delivered in collaboration with interdisciplinary medical staff. J Gastroenterol. (2009) 44:1203–8. 10.1007/s00535-009-0115-x 19728009

[42] Simonelli C Eaton RP. Reduced triglyceride secretion: a metabolic consequence of chronic exercise. Am J Physiol. (1978) 234:E221–7. 10.1152/ajpendo.1978.234.3.E221 629336

[43] Gorski J Oscai LB Palmer WK. Hepatic lipid metabolism in exercise and training. Med Sci Sports Exerc. (1990) 22:213–21. 2192222

[44] Borengasser SJ Rector RS Uptergrove GM Morris EM Perfield JW 2nd Booth FW. Exercise and omega-3 polyunsaturated fatty acid supplementation for the treatment of hepatic steatosis in hyperphagic OLETF rats. J Nutr Metab. (2012) 2012:268680. 10.1155/2012/268680 21918718 PMC3171760

[45] Rector RS Thyfault JP Morris RT Laye MJ Borengasser SJ Booth FW. Daily exercise increases hepatic fatty acid oxidation and prevents steatosis in Otsuka Long-Evans Tokushima Fatty rats. Am J Physiol Gastrointest Liver Physiol. (2008) 294:G619–26. 10.1152/ajpgi.00428.2007 18174272

[46] Rector RS Uptergrove GM Morris EM Borengasser SJ Laughlin MH Booth FW. Daily exercise vs. caloric restriction for prevention of nonalcoholic fatty liver disease in the OLETF rat model. Am J Physiol Gastrointest Liver Physiol. (2011) 300:G874–83. 10.1152/ajpgi.00510.2010 21350190 PMC3094141

[47] Linden MA Fletcher JA Morris EM Meers GM Kearney ML Crissey JM. Combining metformin and aerobic exercise training in the treatment of type 2 diabetes and NAFLD in OLETF rats. Am J Physiol Endocrinol Metab. (2014) 306:E300–10. 10.1152/ajpendo.00427.2013 24326426 PMC3920010

[48] Trefts E Williams AS Wasserman DH. Exercise and the regulation of hepatic metabolism. Prog Mol Biol Transl Sci. (2015) 135:203–25. 10.1016/bs.pmbts.2015.07.010 26477916 PMC4826571

[49] Cartee GD Farrar RP. Exercise training induces glycogen sparing during exercise by old rats. J Appl Physiol. (1985) 64:259–65. 10.1152/jappl.1988.64.1.259 3356644

[50] Podolin DA Pagliassotti MJ Gleeson TT Mazzeo RS. Influence of endurance training on the age-related decline in hepatic glyconeogenesis. Mech Ageing Dev. (1994) 75:81–93. 10.1016/0047-6374(94)90030-2 9128756

[51] Kim KM Lee KS Lee GY Jin H Durrance ES Park HS. Anti-diabetic efficacy of KICG1338, a novel glycogen synthase kinase-3β inhibitor and its molecular characterization in animal models of type 2 diabetes and insulin resistance. Mol Cell Endocrinol. (2015) 409:1–10. 10.1016/j.mce.2015.03.011 25802191

[52] López-Soldado I Zafra D Duran J Adrover A Calbó J Guinovart JJ. Liver glycogen reduces food intake and attenuates obesity in a high-fat diet-fed mouse model. Diabetes (2015) 64:796–807. 10.2337/db14-0728 25277398

[53] Berglund ED Lee-Young RS Lustig DG Lynes SE Donahue EP Camacho RC. Hepatic energy state is regulated by glucagon receptor signaling in mice. J Clin Invest. (2009) 119:2412–22. 10.1172/JCI38650 19662685 PMC2719934

[54] Miller RA Chu Q Xie J Foretz M Viollet B Birnbaum MJ. Biguanides suppress hepatic glucagon signalling by decreasing production of cyclic AMP. Nature (2013) 494:256–60. 10.1038/nature11808 23292513 PMC3573218

[55] Rothman DL Magnusson I Cline G Gerard D Kahn CR Shulman RG. Decreased muscle glucose transport/phosphorylation is an early defect in the pathogenesis of non-insulin-dependent diabetes mellitus. Proc Natl Acad Sci USA. (1995) 92:983–7. 7862678 10.1073/pnas.92.4.983 PMC42621

[56] Rabøl R Petersen KF Dufour S Flannery C Shulman GI. Reversal of muscle insulin resistance with exercise reduces postprandial hepatic de novo lipogenesis in insulin resistant individuals. Proc Natl Acad Sci USA. (2011) 108:13705–9. 10.1073/pnas.1110105108 21808028 PMC3158147

[57] Stallknecht B Vissing J Galbo H. Lactate production and clearance in exercise. Effects of training. A mini-review. Scand J Med Sci Sports (1998) 8:127–31. 10.1111/j.1600-0838.1998.tb00181.x 9659671

[58] Reddy Avula CP Fernandes G. Modulation of antioxidant enzymes and lipid peroxidation in salivary gland and other tissues in mice by moderate treadmill exercise. Aging (1999) 11:246–52. 10.1007/BF03339665 10605613

[59] Venditti P Di Meo S. Effect of training on antioxidant capacity, tissue damage, and endurance of adult male rats. Int J Sports Med. (1997) 18:497–502. 10.1055/s-2007-972671 9414071

[60] Lima TI Monteiro IC Valença S Leal-Cardoso JH Fortunato RS Carvalho DP. Effect of exercise training on liver antioxidant enzymes in STZ-diabetic rats. Life Sci. (2015) 128:64–71. 10.1016/j.lfs.2015.01.031 25744399

[61] Wree A Broderick L Canbay A Hoffman HM Feldstein AE Feldstein, From nafld to nash to cirrhosis-new insights into disease mechanisms. Nat Rev Gastroenterol Hepatol. (2013) 10:627–36. 10.1038/nrgastro.2013.149 23958599

[62] Li Z Murakami T Nakai N Nagasaki M Obayashi M Xu M. Modification by exercise training of activity and enzyme expression of hepatic branched-chain alpha-ketoacid dehydrogenase complex in streptozotocin-induced diabetic rats. J Nutr Sci Vitaminol. (2001) 47:345–50. 10.3177/jnsv.47.345 11814150

[63] Meex RCR Watt MJ. Hepatokines: linking nonalcoholic fatty liver disease and insulin resistance. Nat Rev Endocrinol. (2017) 13:509–20. 10.1038/nrendo.2017.56 28621339

[64] Stefan N Häring HU. The role of hepatokines in metabolism. Nat Rev Endocrinol. (2013) 9:144–52. 10.1038/nrendo.2012.258 23337953

[65] Clemmons DR. Role of insulin-like growth factor in maintaining normal glucose homeostasis. Horm Res. (2004) 62(Suppl.1):77–82. 10.1159/000080763 15761237

[66] Hagström H Stål P Hultcrantz R Brismar K Ansurudeen I. IGFBP-1 and IGF-I as markers for advanced fibrosis in NAFLD - a pilot study. Scand J Gastroenterol. (2017) 52:1427–34. 10.1080/00365521.2017.1379556 28927302

[67] Chishima S Kogiso T Matsushita N Hashimoto E Tokushige K. The Relationship between the growth hormone/insulin-like growth factor system and the histological features of nonalcoholic fatty liver disease. Intern Med. (2017) 56:473–80. 10.2169/internalmedicine.56.7626 28250290 PMC5399195

[68] Zanconato S Moromisato DY Moromisato MY Woods J Brasel JA Leroith D. Effect of training and growth hormone suppression on insulin-like growth factor I mRNA in young rats. J Appl Physiol. (1994) 76:2204–9. 10.1152/jappl.1994.76.5.2204 8063688

[69] Leme JA Silveira RF Gomes RJ Moura RF Sibuya CA Mello MA. Long-term physical training increases liver IGF-I in diabetic rats. Growth Horm IGF Res. (2009) 19:262–6. 10.1016/j.ghir.2008.12.004 19201234

[70] Rojas Vega S Knicker A Hollmann W Bloch W Strüder HK. Effect of resistance exercise on serum levels of growth factors in humans. Horm Metab Res. (2010) 42:982–6. 10.1055/s-0030-1267950 21053157

[71] Dimitridas G Parry-Billings M Bevan S Dunger D Piva T Krause U Effects of insulin-like growth factor-I on the rates of glucose transport and utilization in rat skeletal muscle in vitro. Biochem J. (1992) 285:269–74. 10.1042/bj2850269 1637311 PMC1132776

[72] Dominici FP Cifone D Bartke A Turyn D. Loss of sensitivity to insulin at early events of the insulin signaling pathway in the liver of growth hormone-transgenic mice. J Endocrinol. (1999) 161:383–92. 10.1677/joe.0.1610383 10333541

[73] Demonbreun AR McNally EM. Muscle cell communication in development and repair. Curr Opin Pharmacol. (2017) 34:7–14. 10.1016/j.coph.2017.03.008 28419894 PMC5641474

[74] Schiaffino S Mammucari C. Regulation of skeletal muscle growth by the IGF1-Akt/PKB pathway: insights from genetic models. Skelet Muscle (2011) 1:4. 10.1186/2044-5040-1-4 21798082 PMC3143906

[75] Semsarian C Sutrave P Richmond DR Graham RM. Insulin-like growth factor (IGF-I) induces myotube hypertrophy associated with an increase in anaerobic glycolysis in a clonal skeletal-muscle cell model. Biochem J. (1999) 339:443–51. 10.1042/bj3390443 10191278 PMC1220176

[76] Barton-Davis ER Shoturma DI Musaro A Rosenthal N Sweeney HL. Viral mediated expression of insulin-like growth factor I blocks the aging-related loss of skeletal muscle function. Proc Natl Acad Sci USA. (1998) 95:15603–7. 10.1073/pnas.95.26.15603 9861016 PMC28090

[77] Coleman ME DeMayo F Yin KC Lee HM Geske R Montgomery C. Myogenic vector expression of insulin-like growth factor I stimulates muscle cell differentiation and myofiber hypertrophy in transgenic mice. J Biol Chem. (1995) 270:12109–16. 10.1074/jbc.270.20.12109 7744859

[78] Koo BK Kim D Joo SK Kim JH Chang MS Kim BG. Sarcopenia is an independent risk factor for non-alcoholic steatohepatitis and significant fibrosis. J Hepatol. (2017) 66:123–31. 10.1016/j.jhep.2016.08.019 27599824

[79] Bhanji RA Narayanan P Allen AM Malhi H Watt KD. Sarcopenia in hiding: the risk and consequence of underestimating muscle dysfunction in nonalcoholic steatohepatitis. Hepatology (2017) 66:2055–65. 10.1002/hep.29420 28777879

[80] Kumar KG Trevaskis JL Lam DD Sutton GM Koza RA Chouljenko VN. Identification of adropin as a secreted factor linking dietary macronutrient intake with energy homeostasis and lipid metabolism. Cell Metab. (2008) 8: 468–81. 10.1016/j.cmet.2008.10.011 19041763 PMC2746325

[81] Lovren F Pan Y Quan A Singh KK Shukla PC Gupta M. Adropin is a novel regulator of endothelial function. Circulation (2010) 122 (11 Suppl.):S185–92. 10.1161/CIRCULATIONAHA.109.931782 20837912

[82] Aydin S Kuloglu T Aydin S Eren MN Yilmaz M Kalayci M. Expression of adropin in rat brain, cerebellum, kidneys, heart, liver, and pancreas in streptozotocin-induced diabetes. Mol Cell Biochem. (2013) 380:73–81. 10.1007/s11010-013-1660-4 23620340

[83] Wong CM Wang Y Lee JT Huang Z Wu D Xu A. Adropin is a brain membrane-bound protein regulating physical activity via the NB-3/Notch signaling pathway in mice. J Biol Chem. (2014) 289:25976–86. 10.1074/jbc.M114.576058 25074942 PMC4162195

[84] Fujie S Hasegawa N Sato K Fujita S Sanada K Hamaoka T. Aerobic exercise training-induced changes in serum adropin level are associated with reduced arterial stiffness in middle-aged and older adults. Am J Physiol Heart Circ Physiol. (2015) 309:H1642–7. 10.1152/ajpheart.00338.2015 26371163

[85] Zhang H Jiang L Yang YJ Ge RK Zhou M Hu H. Aerobic exercise improves endothelial function and serum adropin levels in obese adolescents independent of body weight loss. Sci Rep. (2017) 7:17717. 10.1038/s41598-017-18086-3 29255252 PMC5735148

[86] Kersten S Mandard S Tan NS Escher P Metzger D Chambon P. Characterization of the fasting-induced adipose factor FIAF, a novel peroxisome proliferator-activated receptor target gene. J Biol Chem. (2000) 275:28488–93. 10.1074/jbc.M004029200 10862772

[87] Ingerslev B Hansen JS Hoffmann C Clemmesen JO Secher NH Scheler M. Angiopoietin-like protein 4 is an exercise-induced hepatokine in humans, regulated by glucagon and cAMP. Mol Metab. (2017) 6:1286–95. 10.1016/j.molmet.2017.06.018 29031727 PMC5641605

[88] Catoire M Alex S Paraskevopulos N Mattijssen F Evers-van Gogh I Schaart G. Fatty acid-inducible ANGPTL4 governs lipid metabolic response to exercise. Proc Natl Acad Sci USA. (2014) 111:E1043–52. 10.1073/pnas.1400889111 24591600 PMC3964070

[89] Mandard S Zandbergen F van Straten E Wahli W Kuipers F Müller M. The fasting-induced adipose factor/angiopoietin-like protein 4 is physically associated with lipoproteins and governs plasma lipid levels and adiposity. J Biol Chem. (2006) 281:934–44. 10.1074/jbc.M506519200 16272564

[90] Xu A Lam MC Chan KW Wang Y Zhang J Hoo RL. Angiopoietin-like protein 4 decreases blood glucose and improves glucose tolerance but induces hyperlipidemia and hepatic steatosis in mice. Proc Natl Acad Sci USA. (2005) 102:6086–91. 10.1073/pnas.0408452102 15837923 PMC1087912

[91] Staiger H Haas C Machann J Werner R Weisser M Schick F. Muscle-derived angiopoietin-like protein 4 is induced by fatty acids via peroxisome proliferator-activated receptor (PPAR)-δ and is of metabolic relevance in humans. Diabetes (2009) 58:579–89. 10.2337/db07-1438 19074989 PMC2646056

[92] Dijk W Beigneux AP Larsson M Bensadoun A Young SG Kersten S. Angiopoietin-like 4 promotes intracellular degradation of lipoprotein lipase in adipocytes. J Lipid Res. (2016) 57:1670–83. 10.1194/jlr.M067363 27034464 PMC5003152

[93] Yau MH Wang Y Lam KS Zhang J Wu D Xu A. A highly conserved motif within the NH2-terminal coiled-coil domain of angiopoietinlike protein 4 confers its inhibitory effects on lipoprotein lipase by disrupting the enzyme dimerization. J Biol Chem. (2009) 284:11942–52. 10.1074/jbc.M809802200 19246456 PMC2673263

[94] Kersten S Lichtenstein L Steenbergen E Mudde K Hendriks HF Hesselink MK. Caloric restriction and exercise increase plasma ANGPTL4 levels in humans via elevated free fatty acids. Arterioscler Thromb Vasc Biol. (2009) 29:969–74. 10.1161/ATVBAHA.108.182147 19342599

[95] Norheim F Hjorth M Langleite TM Lee S Holen T Bindesbøll C. Regulation of angiopoietin-like protein 4 production during and after exercise. Physiol Rep. (2014) 2:e12109. 10.14814/phy2.12109 25138789 PMC4246580

[96] Bäckhed F Ding H Wang T Hooper LV Koh GY Nagy A. The gut microbiota as an environmental factor that regulates fat storage. Proc Natl Acad Sci USA. (2004) 101:15718–23. 10.1073/pnas.0407076101 15505215 PMC524219

[97] Kahn SM Hryb DJ Nakhla AM Romas NA Rosner W. Sex hormonebinding globulin is synthesized in target cells. J Endocrinol. (2002) 175:113–20. 10.1677/joe.0.1750113 12379495

[98] Ding EL Song Y Malik VS Liu S Sex differences of endogenous sex hormones and risk of type 2 diabetes: a systematic review and metaanalysis. JAMA (2006) 295:1288–99. 10.1001/jama.295.11.1288 16537739

[99] Haffner SM. Sex hormones, obesity, fat distribution, type 2 diabetes and insulin resistance: epidemiological and clinical correlation. Int J Obes Relat Metab Disord. (2000) 24(Suppl. 2):S56–8. 10.1038/sj.ijo.0801279 10997610

[100] Peter A Kantartzis K Machann J Schick F Staiger H Machicao F. Relationships of circulating sex hormone-binding globulin with metabolic traits in humans. Diabetes (2010) 59:3167–73. 10.2337/db10-0179 20841609 PMC2992779

[101] Bonnet F Velayoudom Cephise FL Gautier A Dubois S Massart C Camara A. Role of sex steroids, intrahepatic fat and liver enzymes in the association between SHBG and metabolic features. Clin Endocrinol. (2013) 79:517–22. 10.1111/cen.12089 23121021

[102] Simó R Saez-Lopez C Lecube A Hernandez C Fort JM Selva DM. Adiponectin upregulates SHBG production: molecular mechanisms and potential implications. Endocrinology (2014) 155:2820–30. 10.1210/en.2014-1072 24828613

[103] Simo R Barbosa-Desongles A Hernandez C Selva DM IL1ß down-regulation of sex hormonebinding globulin production by decreasing HNF-4α via MEK-1/2 and JNK MAPK pathways. Mol Endocrinol. (2012) 26:1917–27. 10.1210/me.2012-1152 22902540 PMC5416961

[104] Simo R Barbosa-Desongles A Lecube A Hernandez C Selva DM. Potential role of tumor necrosis factor-α in downregulating sex hormone-binding globulin. Diabetes (2012) 61:372–82. 10.2337/db11-0727 22210320 PMC3266423

[105] Tymchuk CN Tessler SB Barnard RJ. Changes in sex hormone-binding globulin, insulin, and serum lipids in postmenopausal women on a low-fat, high-fiber diet combined with exercise. Nutr Cancer (2000) 38:158–62. 10.1207/S15327914NC382_3 11525592

[106] Kim JW Kim DY. Effects of aerobic exercise training on serum sex hormone binding globulin, body fat index, and metabolic syndrome factors in obese postmenopausal women. Metab Syndr Relat Disord. (2012) 10:452–7. 10.1089/met.2012.0036 22989086

[107] Fahrner CL Hackney AC. Effects of endurance exercise on free testosterone concentration and the binding affinity of sex hormone binding globulin (SHBG). Int J Sports Med. (1998) 19:12–5. 10.1055/s-2007-971872 9506793

[108] Mori K Emoto M Inaba M. Fetuin-A: a multifunctional protein. Recent Pat Endocr Metab Immune Drug Discov. (2011) 5:124–46. 10.2174/187221411799015372 22074587

[109] Denecke B Gräber S Schäfer C Heiss A Wöltje M Jahnen-Dechent W Tissue distribution and activity testing suggest a similar but not identical function of fetuin-B and fetuin-A. Biochem J. (2003) 376:135–45. 10.1042/BJ20030676 12943536 PMC1223762

[110] Auberger P Falquerho L Contreres JO Pages G Le Cam G Rossi B. Characterization of a natural inhibitor of the insulin receptor tyrosine kinase: cDNA cloning, purification, and anti-mitogenic activity. Cell (1989) 58:631–40. 10.1016/0092-8674(89)90098-6 2766355

[111] Mathews ST Chellam N Srinivas PR Cintron VJ Leon MA Goustin AS. α2-HSG, a specific inhibitor of insulin receptor autophosphorylation, interacts with the insulin receptor. Mol Cell Endocrinol. (2000) 164:87–98. 10.1016/S0303-7207(00)00237-9 11026561

[112] Mathews ST Singh GP Ranalletta M Cintron VJ Qiang X Goustin AS. Improved insulin sensitivity and resistance to weight gain in mice null for the Ahsg gene. Diabetes (2002) 51:2450–8. 10.2337/diabetes.51.8.2450 12145157

[113] Pal D Dasgupta S Kundu R Maitra S Das G Mukhopadhyay S. Fetuin-A acts as an endogenous ligand of TLR4 to promote lipid induced insulin resistance. Nat Med. (2012) 18:1279–85. 10.1038/nm.2851 22842477

[114] Stefan N Haring HU. Circulating fetuin-A and free fatty acids interact to predict insulin resistance in humans. Nat Med. (2013) 19:394–5. 10.1038/nm.3116 23558619

[115] Zhang LY Liu T Teng YQ Yao XY Zhao TT Lin LY Effect of a 12-week aerobic exercise training on serum fetuin-A and adipocytokine levels in type 2 diabetes. Exp Clin Endocrinol Diabetes (2017) 125:1–6. 10.1055/s-0043-115904 28750433

[116] Malin SK del Rincon JP Huang H Kirwan JP. Exercise-induced lowering of fetuin-A may increase hepatic insulin sensitivity. Med Sci Sports Exerc. (2014) 46:2085–90. 10.1249/MSS.0000000000000338 24637346 PMC4640446

[117] Malin SK Mulya A Fealy CE Haus JM Pagadala MR Scelsi AR. Fetuin-A is linked to improved glucose tolerance after short-term exercise training in nonalcoholic fatty liver disease. J Appl Physiol. (2013) 115:988–94. 10.1152/japplphysiol.00237.2013 23928114 PMC3798818

[118] Winn NC Liu Y Rector RS Parks EJ Ibdah JA Kanaley JA Energy-matched moderate and high intensity exercise training improves nonalcoholic fatty liver disease risk independent of changes in body mass or abdominal adiposity - A randomized trial. Metabolism (2018) 78:128–140. 10.1016/j.metabol.2017.08.012 28941598

[119] Sargeant JA Aithal GP Takamura T Misu H Takayama H Douglas JA. The influence of adiposity and acute exercise on circulating hepatokines in normal-weight and overweight/obese men. Appl Physiol Nutr Metab. (2018) 43:482–90. 10.1139/apnm-2017-0639 29220580

[120] Matsumoto Y Adams V Jacob S Mangner N Schuler G Linke A. Regular exercise training prevents aortic valve disease in low-density lipoprotein-receptor-deficient mice. Circulation (2010) 121:759–67. 10.1161/CIRCULATIONAHA.109.892224 20124122

[121] Meex RC Hoy AJ Morris A Brown RD Lo JC Burke M. Fetuin B is a secreted hepatocyte factor linking steatosis to impaired glucose metabolism. Cell Metab. (2015) 22:1078–89. 10.1016/j.cmet.2015.09.023 26603189

[122] Hara H Uchida S Yoshimura H Aoki M Toyoda Y Sakai Y. Isolation and characterization of a novel liver-specific gene, hepassocin, upregulated during liver regeneration. Biochim Biophys Acta (2000) 1492:31–44. 10.1016/S0167-4781(00)00056-7 11004478

[123] Li CY Cao CZ Xu WX Cao MM Yang F Dong L. Recombinant human hepassocin stimulates proliferation of hepatocytes *in vivo* and improves survival in rats with fulminant hepatic failure. Gut (2010) 59:817–26. 10.1136/gut.2008.171124 19880967

[124] Cao MM Xu WX Li CY Cao CZ Wang ZD Yao JW. Hepassocin regulates cell proliferation of the human hepatic cells L02 and hepatocarcinoma cells through different mechanisms. J Cell Biochem. (2011) 112:2882–90. 10.1002/jcb.23202 21618590

[125] Hara H Yoshimura H Uchida S Toyoda Y Aoki M Sakai Y. Molecular cloning and functional expression analysis of a cDNA for human hepassocin, a liver-specific protein with hepatocyte mitogenic activity. Biochim Biophys Acta (2001) 1520:45–53. 10.1016/S0167-4781(01)00249-4 11470158

[126] Wu HT Ou HY Hung HC Su YC Lu FH Wu JS. A novel hepatokine, HFREP1, plays a crucial role in the development of insulin resistance and type 2 diabetes. Diabetologia (2016) 59:1732–42. 10.1007/s00125-016-3991-7 27221093

[127] Sreekumar R Rosado B Rasmussen D Charlton M. Hepatic gene expression in histologically progressive nonalcoholic steatohepatitis. Hepatology (2003) 38:244–51. 10.1053/jhep.2003.50290 12830008

[128] Yamagoe S Mizuno S Suzuki K. Molecular cloning of human and bovine LECT2 having a neutrophil chemotactic activity and its specific expression in the liver. Biochim Biophys Acta (1998) 1396:105–13. 10.1016/S0167-4781(97)00181-4 9524238

[129] Yamagoe S Yamakawa Y Matsuo Y Minowada J Mizuno S Suzuki K. Purification and primary amino acid sequence of a novel neutrophil chemotactic factor LECT2. Immunol Lett (1996) 52:9–13. 10.1016/0165-2478(96)02572-2 8877413

[130] Lan F Misu H Chikamoto K Takayama H Kikuchi A Mohri K. LECT2 functions as a hepatokine that links obesity to skeletal muscle insulin resistance. Diabetes (2014) 63:1649–64. 10.2337/db13-0728 24478397

[131] Rydén M. Fibroblast growth factor 21: an overview from a clinical perspective. Cell Mol Life Sci. (2009) 66:2067–73. 10.1007/s00018-009-0003-9 19277467 PMC11115664

[132] Kharitonenkov A Wroblewski VJ Koester A Chen YF Clutinger CK Tigno XT. The metabolic state of diabetic monkeys is regulated by fibroblast growth factor-21. Endocrinology (2007) 148:774–81. 10.1210/en.2006-1168 17068132

[133] Bostrom P Wu J Jedrychowski MP Korde A Ye L Lo JC. A PGC1-alpha-dependent myokine that drives brown-fat-like development of white fat and thermogenesis. Nature (2012) 481:463–8. 10.1038/nature10777 22237023 PMC3522098

[134] Fisher FM Kleiner S Douris N Fox EC Mepani RJ Verdeguer F. FGF21 regulates PGC-1α and browning of white adipose tissues in adaptive thermogenesis. Genes Dev. (2012) 26:271–81. 10.1101/gad.177857.111 22302939 PMC3278894

[135] Ding X Boney-Montoya J Owen BM Bookout AL Coate KC Mangelsdorf DJ. βKlotho is required for fibroblast growth factor 21 effects on growth and metabolism. Cell Metab. (2012) 16:387–93. 10.1016/j.cmet.2012.08.002 22958921 PMC3447537

[136] Kim KH Kim SH Min YK Yang HM Lee JB Lee MS. Acute exercise induces FGF21 expression in mice and in healthy humans. PLoS ONE (2013) 8:e63517. 10.1371/journal.pone.0063517 23667629 PMC3646740

[137] Fletcher JA Linden MA Sheldon RD Meers GM Morris EM Butterfield A. Fibroblast growth factor 21 and exercise-induced hepatic mitochondrial adaptations. Am J Physiol Gastrointest Liver Physiol. (2016) 310:G832–43. 10.1152/ajpgi.00355.2015 27012775 PMC4895870

[138] Yang Q Graham TE Mody N Preitner F Peroni OD Zabolotny JM. Serum retinol binding protein 4 contributes to insulin resistance in obesity and type 2 diabetes. Nature (2005) 436:356–62. 10.1038/nature03711 16034410

[139] Blaner WS. Retinol-binding protein: the serum transport protein for vitamin A. Endocr Rev. (1989) 10:308–16. 10.1210/edrv-10-3-308 2550213

[140] Graham TE Yang Q Blüher M Hammarstedt A Ciaraldi TP Henry RR. Retinol-binding protein 4 and insulin resistance in lean, obese, and diabetic subjects. N Engl J Med. (2006) 354:2552–63. 10.1056/NEJMoa054862 16775236

[141] Moraes-Vieira PM Yore MM Dwyer PM Syed I Aryal P Kahn BB. RBP4 activates antigenpresenting cells, leading to adipose tissue inflammation and systemic insulin resistance. Cell Metab. (2014) 19:512–26. 10.1016/j.cmet.2014.01.018 24606904 PMC4078000

[142] Haider DG Schindler K Prager G Bohdjalian A Luger A Wolzt M. Serum retinol-binding protein 4 is reduced after weight loss in morbidly obese subjects. J Clin Endocrinol Metab. (2007) 92:1168–71. 10.1210/jc.2006-1839 17164313

[143] Lee JW Lee HR Shim JY Im JA Lee DC. Abdominal visceral fat reduction is associated with favorable changes of serum retinol binding protein-4 in nondiabetic subjects. Endocr J. (2008) 55:811–8. 10.1507/endocrj.K08E-030 18493106

[144] Ku YH Han KA Ahn H Kwon H Koo BK Kim HC Resistance exercise did not alter intramuscular adipose tissue but reduced retinol-binding protein-4 concentration in individuals with type 2 diabetes mellitus. J Int Med Res. (2010) 38:782–91. 10.1177/147323001003800305 20819415

[145] Lim S Choi SH Jeong IK Kim JH Moon MK Park KS. Insulin-sensitizing effects of exercise on adiponectin and retinol-binding protein-4 concentrations in young and middle-aged women. J Clin Endocrinol Metab. (2008) 93:2263–8. 10.1210/jc.2007-2028 18334592

[146] Mansouri M Keshtkar A Hasani-Ranjbar S Soleymani Far E Tabatabaei-Malazy O Omidfar K. The impact of one session resistance exercise on plasma adiponectin and RBP4 concentration in trained and untrained healthy young men. Endocr J. (2011) 58:861–8. 10.1507/endocrj.EJ11-0046 21836369

[147] Marschner RA Pinto G Borges J Markoski MM Schaan BD Lehnen AM Short-Term Detraining does not Change Insulin Sensitivity and RBP4 in Rodents Previously Submitted to Aerobic Exercise. Horm Metab Res. (2017) 49:58–63. 10.1055/s-0042-115176 27589346

[148] Mansouri M Nikooie R Keshtkar A Larijani B Omidfar K. Effect of endurance training on retinol-binding protein 4 gene expression and its protein level in adipose tissue and the liver in diabetic rats induced by a high-fat diet and streptozotocin. J Diabetes Investig. (2014) 5:484–91. 10.1111/jdi.12186 25411614 PMC4188104

[149] Misu H Takamura T Takayama H Hayashi H Matsuzawa-Nagata N Kurita S. A liver-derived secretory protein, selenoprotein P, causes insulin resistance. Cell Metab. (2010) 12:483–95. 10.1016/j.cmet.2010.09.015 21035759

[150] Misu H Takayama H Saito Y Mita Y Kikuchi A Ishii KA. Deficiency of the hepatokine selenoprotein P increases responsiveness to exercise in mice through upregulation of reactive oxygen species and AMP-activated protein kinase in muscle. Nat Med. (2017) 23:508–16. 10.1038/nm.4295 28263310

[151] Pograjc L Stibilj V Falnoga I. Impact of intensive physical activity on selenium status. Biol Trace Elem Res. (2012) 145:291–9. 10.1007/s12011-011-9204-9 21960354

[152] Stanford KI Middelbeek RJ Goodyear LJ Exercise effects on white adipose tissue: beiging and metabolic adaptations. Diabetes (2015) 64:2361–8. 10.2337/db15-0227 26050668 PMC4477356

[153] Yu N Ruan Y Gao X Sun J. Systematic review and meta-analysis of randomized, controlled trials on the effect of exercise on serum leptin and adiponectin in overweight and obese individuals. Horm Metab Res. (2017) 49:164–73. 10.1055/s-0042-121605 28249299

[154] Kamohara S Burcelin R Halaas JL Friedman JM Charron MJ. Acute stimulation of glucose metabolism in mice by leptin treatment. Nature (1997) 389:374–7. 9311777 10.1038/38717

[155] Shimomura I Hammer RE Ikemoto S Brown MS Goldstein JL. Leptin reverses insulin resistance and diabetes mellitus in mice with congenital lipodystrophy. Nature (1999) 401:73–76. 10485707 10.1038/43448

[156] Owecki M Nikisch E Miczke A Pupek-Musialik D Sowinski J. Leptin soluble leptin receptors, free leptin index, and their relationship with insulin resistance and BMI: high normal BMI is the threshold for serum leptin increase in humans. Horm Metab Res. (2010) 42:585–9. 10.1055/s-0030-1253422 20455195

[157] Crujeiras AB Carreira MC Cabia B Andrade S Amil M Casanueva FF. Leptin resistance in obesity: An epigenetic landscape. Life Sci. (2015) 140:57–63. 10.1016/j.lfs.2015.05.003 25998029

[158] Wang J Leclercq I Brymora JM Xu N Ramezani-Moghadam M London RM. Kupffer cells mediate leptin-induced liver fibrosis. Gastroenterology (2009) 137:713–23. 10.1053/j.gastro.2009.04.011 19375424 PMC2757122

[159] Lanthier N Horsmans Y Leclercq IA. The metabolic syndrome: how it may influence hepatic stellate cell activation and hepatic fibrosis. Curr Opin Clin Nutr Metab Care (2009) 12:404–11. 10.1097/MCO.0b013e32832c7819 19474722

[160] Arita Y Kihara S Ouchi N Takahashi M Maeda K Miyagawa J. Paradoxical decrease of an adipose-specific protein, adiponectin, in obesity. Biochem Biophys Res Commun. (1999) 257:79–83. 10092513 10.1006/bbrc.1999.0255

[161] Matsubara M Maruoka S Katayose S. Inverse relationship between plasma adiponectin and leptin concentrations in normal-weight and obese women. Eur J Endocrinol. (2002) 147:173–80. 10.1530/eje.0.1470173 12153737

[162] Zietz B Herfarth H Paul G Ehling A Müller-Ladner U Schölmerich J. Adiponectin represents an independent cardiovascular risk factor predicting serum HDL cholesterol levels in type 2 diabetes. FEBS Lett. (2003) 545:103–4. 10.1016/S0014-5793(03)00568-4 12804757

[163] Fruebis J Tsao TS Javorschi S Ebbets-Reed D Erickson MR Yen FT. Proteolytic cleavage product of 30-kDa adipocyte complement-related protein increases fatty acid oxidation in muscle and causes weight loss in mice. Proc Natl Acad Sci USA. (2001) 98:2005–10. 10.1073/pnas.98.4.2005 11172066 PMC29372

[164] Buechler C Wanninger J Neumeier M. Adiponectin, a key adipokine in obesity related liver diseases. World J Gastroenterol. (2011) 17:2801–11. 10.3748/wjg.v17.i23.2801 21734787 PMC3120939

[165] Awazawa M Ueki K Inabe K Yamauchi T Kaneko K Okazaki Y. Adiponectin suppresses hepatic SREBP1c expression in an AdipoR1/LKB1/AMPK dependent pathway. Biochem Biophys Res Commun. (2009) 382:51–6. 10.1016/j.bbrc.2009.02.131 19254698

[166] Kamada Y Takehara T Hayashi N. Adipocytokines and liver disease. J Gastroenterol. (2008) 43:811–22. 10.1007/s00535-008-2213-6 19012034

[167] Handa P Maliken BD Nelson JE Morgan-Stevenson V Messner DJ Dhillon BK. Reduced adiponectin signaling due to weight gain results in nonalcoholic steatohepatitis through impaired mitochondrial biogenesis. Hepatology (2014) 60:133–45. 10.1002/hep.26946 24464605 PMC5993561

[168] Francque S Verrijken A Caron S Prawitt J Paumelle R Derudas B. PPARα gene expression correlates with severity and histological treatment response in patients with non-alcoholic steatohepatitis. J Hepatol. (2015) 63:164–73. 10.1016/j.jhep.2015.02.019 25703085

[169] van der Poorten D Samer CF Ramezani-Moghadam M Coulter S Kacevska M Schrijnders D. Hepatic fat loss in advanced nonalcoholic steatohepatitis: are alterations in serum adiponectin the cause? Hepatology (2013) 57:2180–8. 10.1002/hep.26072 22996622

[170] Matsumoto H Tamura S Kamada Y Kiso S Fukushima J Wada A. Adiponectin deficiency exacerbates lipopolysaccharide/D-galactosamine-induced liver injury in mice. World J Gastroenterol. (2006) 12:3352–8. 10.3748/wjg.v12.i21.3352 16733851 PMC4087865

[171] Tsao TS Murrey HE Hug C Lee DH Lodish HF. Oligomerization state-dependent activation of NF-kappa B signaling pathway by adipocyte complement-related protein of 30 kDa (Acrp30). J Biol Chem. (2002) 277:29359–62. 10.1074/jbc.C200312200 12087086

[172] Park PH McMullen MR Huang H Thakur V Nagy LE. Short-term treatment of RAW264.7 macrophages with adiponectin increases tumor necrosis factor-alpha (TNF-alpha) expression via ERK1/2 activation and Egr-1 expression: role of TNF-alpha in adiponectin-stimulated interleukin-10 production. J Biol Chem. (2007) 282:21695–703. 10.1074/jbc.M701419200 17537727 PMC1978175

[173] Tomita K Oike Y Teratani T Taguchi T Noguchi M Suzuki T. Hepatic AdipoR2 signaling plays a protective role against progression of nonalcoholic steatohepatitis in mice. Hepatology (2008) 48:458–73. 10.1002/hep.22365 18666257

[174] Ding X Saxena NK Lin S Xu A Srinivasan S Anania FA. The roles of leptin and adiponectin: a novel paradigm in adipocytokine regulation of liver fibrosis and stellate cell biology. Am J Pathol. (2005) 166:1655–69. 10.1016/S0002-9440(10)62476-5 15920151 PMC1602420

[175] Handy JA Saxena NK Fu P Lin S Mells JE Gupta NA. Adiponectin activation of AMPK disrupts leptin-mediated hepatic fibrosis via suppressors of cytokine signaling (SOCS-3). J Cell Biochem. (2010) 110:1195–207. 10.1002/jcb.22634 20564215 PMC2907429

[176] Harris LA Skinner JR Shew TM Pietka TA Abumrad NA Wolins NE. Perilipin 5-driven lipid droplet accumulation in skeletal muscle stimulates the expression of fibroblast growth factor 21. Diabetes (2015) 64:2757–68. 10.2337/db14-1035 25829453 PMC4512215

[177] Febbraio MA Pedersen B. Muscle-derived interleukin-6: mechanisms for activation and possible biological roles. FASEB J. (2002) 16:1335–47. 10.1096/fj.01-0876rev 12205025

[178] Pedersen BK Steensberg A Schjerling P. Muscle-derived interleukin-6: possible biological effects. J Physiol. (2001) 536(Pt 2):329–37. 10.1111/j.1469-7793.2001.0329c.xd 11600669 PMC2278876

[179] Pedersen BK Febbraio MA. Muscle as an endocrine organ: focus on muscle-derived interleukin-6. Physiol Rev. (2008) 88:1379–406. 10.1152/physrev.90100.2007 18923185

[180] van Hall G Steensberg A Sacchetti M Fischer C Keller C Schjerling P. Interleukin-6 stimulates lipolysis and fat oxidation in humans. J Clin Endocrinol Metab. (2003) 88:3005–10. 10.1210/jc.2002-021687 12843134

[181] Bouzakri K Plomgaard P Berney T Donath MY Pedersen BK Halban PA. Bimodal effect on pancreatic β-cells of secretory products from normal or insulinresistant human skeletal muscle. Diabetes (2011) 60:1111–21. 10.2337/db10-1178 21378173 PMC3064085

[182] Gopurappilly R Bhonde R. Can multiple intramuscular injections of mesenchymal stromal cells overcome insulin resistance offering an alternative mode of cell therapy for type 2 diabetes? Med Hypotheses (2012) 78:393–5. 10.1016/j.mehy.2011.11.021 22192909

[183] Febbraio MA Hiscock N Sacchetti M Fischer CP Pedersen BK. Interleukin-6 is a novel factor mediating glucose homeostasis during skeletal muscle contraction. Diabetes (2004) 53:1643–1648. 10.2337/diabetes.53.7.1643 15220185

[184] Banzet S Koulmann N Simler N Sanchez H Chapot R Serrurier B. Control of gluconeogenic genes during intense/prolonged exercise: hormoneindependent effect of muscle-derived IL-6 on hepatic tissue and PEPCK mRNA. J Appl Physiol. (2009) 107:1830–9. 10.1152/japplphysiol.00739.2009 19850730

[185] Nehlsen-Cannarella SL Fagoaga OR Nieman DC Henson DA Butterworth DE Schmitt RL. Carbohydrate and the cytokine response to 2.5 h of running. J Appl Physiol. (1997) 82:1662–7. 9134917 10.1152/jappl.1997.82.5.1662

[186] Pedersen L Hojman P. Muscle-to-organ cross talk mediated by myokines. Adipocyte (2012) 1:164–7. 10.4161/adip.20344 23700527 PMC3609091

[187] Selzner M Camargo CA Clavien PA. Ischemia impairs liver regeneration after major tissue loss in rodents: protective effects of interleukin-6. Hepatology (1999) 30:469–75. 10421656 10.1002/hep.510300215

[188] Hong F Radaeva S Pan HN Tian Z Veech R Gao B. Interleukin 6 alleviates hepatic steatosis and ischemia/reperfusion injury in mice with fatty liver disease. Hepatology (2004) 40:933–41. 10.1002/hep.20400 15382116

[189] Camargo CA Jr Madden JF Gao W Selvan RS Clavien PA. Interleukin-6 protects liver against warm ischemia/reperfusion injury and promotes hepatocyte proliferation in the rodent. Hepatology (1997) 26:513–20. 9397992 10.1002/hep.510260619

[190] Streetz KL Tacke F Leifeld L Wüstefeld T Graw A Klein C. Interleukin 6/gp130-dependent pathways are protective during chronic liver diseases. Hepatology (2003) 38:218–29. 10.1053/jhep.2003.50268 12830005

[191] Vida M Serrano A Romero-Cuevas M Pavón FJ González-Rodriguez A Gavito AL. IL-6 cooperates with peroxisome proliferator-activated receptor-α-ligands to induce liver fatty acid binding protein (LFABP) up-regulation. Liver Int. (2013) 33:1019–28. 10.1111/liv.12156 23534555

[192] Varga T Czimmerer Z Nagy L. PPARs are a unique set of fatty acid regulated transcription factors controlling both lipid metabolism and inflammation. Biochim Biophys Acta (2011) 1812:1007–22. 10.1016/j.bbadis.2011.02.014 21382489 PMC3117990

[193] Senn JJ Klover PJ Nowak IA Mooney RA. Interleukin-6 induces cellular insulin resistance in hepatocytes. Diabetes (2002) 51:3391–9. 10.2337/diabetes.51.12.3391 12453891

[194] Donath MY Shoelson SE. Type 2 diabetes as an inflammatory disease. Nat Rev Immunol. (2011) 11:98–107. 10.1038/nri2925 21233852

[195] Park EJ Lee JH Yu GY He G Ali SR Holzer RG. Dietary and genetic obesity promote liver inflammation and tumorigenesis by enhancing IL-6 and TNF expression. Cell (2010) 140:197–208. 10.1016/j.cell.2009.12.052 20141834 PMC2836922

[196] Wallenius V Wallenius K Ahrén B Rudling M Carlsten H Dickson SL. Interleukin-6-deficient mice develop mature-onset obesity. Nat Med. (2002) 8:75–9. 10.1038/nm0102-75 11786910

[197] Abd El-Kader SM Al-Jiffri OH Al-Shreef FM Markers of liver function and inflammatory cytokines modulation by aerobic versus resisted exercise training for nonalcoholic steatohepatitis patients. Afr Health Sci. (2014) 14:551–7. 10.4314/ahs.v14i3.8 25352871 PMC4209634

[198] Askari H Rajani SF Poorebrahim M Haghi-Aminjan H Raeis-Abdollahi E Abdollahi M. A glance at the therapeutic potential of irisin against diseases involving inflammation, oxidative stress, and apoptosis: an introductory review. Pharmacol Res. (2018) 129:44–55. 10.1016/j.phrs.2018.01.012 29414191

[199] Park MJ Kim DI Choi JH Heo YR Park SH. New role of irisin in hepatocytes: The protective effect of hepatic steatosis *in vitro*. Cell Signal (2015) 27:1831–9. 10.1016/j.cellsig.2015.04.010 25917316

[200] Choi ES Kim MK Song MK Kim JM Kim ES Chung WJ. Association between serum irisin levels and non-alcoholic fatty liver disease in health screen examinees. PLoS ONE (2014) 9:e110680. 10.1371/journal.pone.0110680 25343462 PMC4208808

[201] Batirel S Bozaykut P Mutlu Altundag E Kartal Ozer N Mantzoros CS. Mantzoros, The effect of irisin on antioxidant system in liver. Free Radic Biol Med. (2014) 75 (Suppl.1):S16. 10.1016/j.freeradbiomed.2014.10.592 26461295

[202] Samy DM Ismail CA Nassra RA. Circulating irisin concentrations in rat models of thyroid dysfunction – effect of exercise. Metabolism (2015) 64:804–13. 10.1016/j.metabol.2015.01.001 25720940

[203] Ohde D Brenmoehl J Walz C Tuchscherer A Wirthgen E Hoeflich A. Comparative analysis of hepatic miRNA levels in male marathon mice reveals a link between obesity and endurance exercise capacities. J Comp Physiol B (2016) 186:1067–78. 10.1007/s00360-016-1006-0 27278158

[204] Goedeke L Salerno A Ramírez CM Guo L Allen RM Yin X. Long-term therapeutic silencing of miR-33 increases circulating triglyceride levels and hepatic lipid accumulation in mice. EMBO Mol Med. (2014) 6:1133–41. 10.15252/emmm.201404046 25038053 PMC4197861

[205] Ghareghani P Shanaki M Ahmadi S Khoshdel AR Rezvan N Meshkani R. Aerobic endurance training improves nonalcoholic fatty liver disease (NAFLD) features via miR-33 dependent autophagy induction in high fat diet fed mice. Obes Res Clin Pract. (2018) 12:80–9. 10.1016/j.orcp.2017.01.004 28163011

[206] Xiao J Bei Y Liu J Dimitrova-Shumkovska J Kuang D Zhou Q. miR-212 downregulation contributes to the protective effect of exercise against non-alcoholic fatty liver via targeting FGF-21. J Cell Mol Med. (2016) 20:204–16. 10.1111/jcmm.12733 26648452 PMC4727558

[207] Saltzman ET Palacios T Thomsen M Vitetta L. Intestinal microbiome shifts, dysbiosis, inflammation, and non-alcoholic fatty liver disease. Front Microbiol. (2018) 9:61. 10.3389/fmicb.2018.00061 29441049 PMC5797576

[208] Loomba R Seguritan V Li W Long T Klitgord N Bhatt A. Gut microbiome-based metagenomic signature for non-invasive detection of advanced fibrosis in human nonalcoholic fatty liver disease. Cell Metab. (2017) 25:1054–62. 10.1016/j.cmet.2017.04.001 28467925 PMC5502730

[209] Panasevich MR Peppler WT Oerther DB Wright DC Rector RS. Microbiome and NAFLD: potential influence of aerobic fitness and lifestyle modification. Physiol Genomics (2017) 49:385–99. 10.1152/physiolgenomics.00012.2017 28600319

[210] Imajo K Fujita K Yoneda M Nozaki Y Ogawa Y Shinohara Y. Hyperresponsivity to low-dose endotoxin during progression to nonalcoholic steatohepatitis is regulated by leptin-mediated signaling. Cell Metab. (2012) 16:44–54. 10.1016/j.cmet.2012.05.012 22768838

[211] Luther J Garber JJ Khalili H Dave M Bale SS Jindal R. Hepatic injury in nonalcoholic steatohepatitis contributes to altered intestinal permeability. Cell Mol Gastroenterol Hepatol. (2015) 1:222–32. 10.1016/j.jcmgh.2015.01.001 26405687 PMC4578658

[212] Henao-Mejia J Elinav E Jin C Hao L Mehal WZ Strowig T. Inflammasome-mediated dysbiosis regulates progression of NAFLD and obesity. Nature (2012) 482:179–85. 10.1038/nature10809 22297845 PMC3276682

[213] Zhu L Baker SS Gill C Liu W Alkhouri R Baker RD Characterization of gut microbiome in nonalcoholic steatohepatitis (NASH) patients: a connection between endogenous alcohol and NASH. Hepatology (2013) 57:601–9. 10.1002/hep.26093 23055155

[214] Allen JM Berg Miller ME Pence BD Whitlock K Nehra V Gaskins HR. Voluntary and forced exercise differentially alters the gut microbiome in C57BL/6J mice. J Appl Physiol. (2015) 118:1059–66. 10.1152/japplphysiol.01077.2014 25678701

[215] Denou E Marcinko K Surette MG Steinberg GR Schertzer JD. High-intensity exercise training increases the diversity and metabolic capacity of the mouse distal gut microbiota during diet-induced obesity. Am J Physiol Endocrinol Metab. (2016) 310:E982–93. 10.1152/ajpendo.00537.2015 27117007 PMC4935139

[216] Sweeney TE Morton JM. The human gut microbiome: a review of the effect of obesity and surgically induced weight loss. JAMA Surg. (2013) 148:563–9. 10.1001/jamasurg.2013.5 23571517 PMC4392891

[217] Evans CC LePard KJ Kwak JW Stancukas MC Laskowski S Dougherty J. Exercise prevents weight gain and alters the gut microbiota in a mouse model of high fat diet-induced obesity. PLoS ONE (2014) 9:e92193. 10.1371/journal.pone.0092193 24670791 PMC3966766

[218] Kang SS Jeraldo PR Kurti A Miller ME Cook MD Whitlock K. Diet and exercise orthogonally alter the gut microbiome and reveal independent associations with anxiety and cognition. Mol Neurodegener. (2014) 9:36. 10.1186/1750-1326-9-36 25217888 PMC4168696

[219] Cani PD Neyrinck AM Fava F Knauf C Burcelin RG Tuohy KM. Selective increases of bifidobacteria in gut microflora improve high-fat-diet-induced diabetes in mice through a mechanism associated with endotoxaemia. Diabetologia (2007) 50:2374–83. 10.1007/s00125-007-0791-0 17823788

[220] Cani PD Possemiers S Van de Wiele T Guiot Y Everard A Rottier O. Changes in gut microbiota control inflammation in obese mice through a mechanism involving GLP-2-driven improvement of gut permeability. Gut (2009) 58:1091–103. 10.1136/gut.2008.165886 19240062 PMC2702831

[221] Campbell SC Wisniewski PJ Noji M McGuinness LR Häggblom MM Lightfoot SA. The effect of diet and exercise on intestinal integrity and microbial diversity in mice. PLoS ONE (2016) 11:e0150502. 10.1371/journal.pone.0150502 26954359 PMC4783017

[222] Angulo P Kleiner DE Dam-Larsen S Adams LA Bjornsson ES Charatcharoenwitthaya P. Liver fibrosis, but no other histologic features, is associated with long-term outcomes of patients with nonalcoholic fatty liver disease. Gastroenterology (2015) 149:389–97. 10.1053/j.gastro.2015.04.043 25935633 PMC4516664

[223] Dulai PS Singh S Patel J Soni M Prokop LJ Younossi Z. Increased risk of mortality by fibrosis stage in nonalcoholic fatty liver disease: systematic review and meta-analysis. Hepatology (2017) 65:1557–65. 10.1002/hep.29085 28130788 PMC5397356

[224] Kistler KD Brunt EM Clark JM Diehl AM Sallis JF Schwimmer JB. Physical activity recommendations, exercise intensity, and histological severity of nonalcoholic fatty liver disease. Am J Gastroenterol. (2011) 106:460–8. 10.1038/ajg.2010.488 21206486 PMC3070294

[225] Dulai PS Sirlin CB Loomba R. MRI and MRE for non-invasive quantitative assessment of hepatic steatosis and fibrosis in NAFLD and NASH: Clinical trials to clinical practice. J Hepatol. (2016) 65:1006–16. 10.1016/j.jhep.2016.06.005 27312947 PMC5124376

[226] Imajo K Kessoku T Honda Y Tomeno W Ogawa Y Mawatari H. Magnetic resonance imaging more accurately classifies steatosis and fibrosis in patients with nonalcoholic fatty liver disease than transient elastography. Gastroenterology (2016) 150:626–37. 10.1053/j.gastro.2015.11.048 26677985

[227] Colombo M Gregersen S Kruhoeffer M Agger A Xiao J Jeppesen PB. Prevention of hyperglycemia in Zucker diabetic fatty rats by exercise training: effects on gene expression in insulin-sensitive tissues determined by high-density oligonucleotide microarray analysis. Metabolism (2005) 54:1571–81. 10.1016/j.metabol.2005.06.003 16311088

[228] Lee KY Kim SJ Cha YS So JR Park JS Kang KS. Effect of exercise on hepatic gene expression in an obese mouse model using cDNA microarrays. Obesity (2006) 14:1294–302. 10.1038/oby.2006.147 16988071

[229] Aoi W Ichiishi E Sakamoto N Tsujimoto A Tokuda H Yoshikawa T. Effect of exercise on hepatic gene expression in rats: a microarray analysis. Life Sci. (2004) 75:3117–28. 10.1016/j.lfs.2004.04.053 15488892

[230] Catoire M Mensink M Kalkhoven E Schrauwen P Kersten S. Identification of human exercise-induced myokines using secretome analysis. Physiol Genomics (2014) 46:256–67. 10.1152/physiolgenomics.00174.2013 24520153

