# Supplementary material for: Therapeutic Approaches to Nonalcoholic Fatty Liver Disease: Exercise Intervention and Related Mechanisms
Source: Front Endocrinol (Lausanne). 2018 Oct 15;9:588. doi: 10.3389/fendo.2018.00588 (PMC6196235; doi:10.3389/fendo.2018.00588)
Supplement: Supplementary file 1 [file Table_1.DOCX]

Supplementary material 1

Protocols of exercise training on NAFLD

| Author (reference) | Design | n | Age (year) | Female (n) | BMI | Dietary consulting |
| --- | --- | --- | --- | --- | --- | --- |
| Bacchi et al (19) | RCT | 14 | 55.6 | 10 | 30.5 | Yes |
| Bacchi et al (19) | RCT | 17 | 56 | 5 | 28.8 | Yes |
| Bhat et al (18) | N-RCT | 45 | 40.1 | 8 | 26.7 | Yes |
| Cassidy et al (29) | RCT | 12 | 61 | 2 | 31.5 | No |
| Chen et al (12) | N-RCT | 16 | 40.1 | 6 | 30.2 | Yes |
| Chen et al (12) | N-RCT | 23 | 36 | 7 | 30.7 | No |
| Cuthbertson et al (30) | RCT | 30 | 50 | 7 | 30.6 | No |
| Fealy et al (28) | SAT | 13 | 58 | NA | 35.2 | No |
| Hallsworth et al (25) | RCT | 12 | 54 | NA | 31 | No |
| Haus et al (20) | SAT | 17 | 54 | NA | 34.4 | No |
| Houghton et al (31) | RCT | 12 | 54 | NA | 33 | No |
| Johnson et al (14) | RCT | 12 | 49.1 | NA | 32.2 | No |
| Kantartzis et al (13) | SAT | 50 | 47.4 | 22 | 31.5 | Yes |
| Keating et al (24) | N-RCT | 12 | 44.2 | 6 | 36.3 | No |
| Keating et al (24) | RCT | 12 | 39.1 | 9 | 32.2 | No |
| Keating et al (24) | N-RCT | 12 | 45.5 | 7 | 33.9 | No |
| Keating et al (24) | N-RCT | 12 | 45.6 | 9 | 31.3 | No |
| Khaoshbaten et al (21) | N-RCT | 45 | 35.6 | 16 | 28.9 | No |
| Lee et al (27) | RCT | 16 | 14.6 | 0 | 34.5 | Yes |
| Lee et al (27) | RCT | 16 | 15.2 | 0 | 33.6 | Yes |
| Oh et al (23) | CaseCT | 40 | 52.6 | 0 | 29.4 | Yes |
| Oh et al (23) | CaseCT | 42 | 49 | 0 | 28.8 | Yes |
| Pugh et al (35) | SAT | 34 | 48 | 12 | 31 | No |
| Rezende et al (32) | RCT | 19 | 56.2 | 19 | 34.1 | No |
| Shamsoddini et al (26) | RCT | 10 | 45.9 | 0 | 30.6 | No |
| Shamsoddini et al (26) | RCT | 10 | 39.7 | 0 | 28.1 | No |
| Shojaee-Moradie et al (33) | RCT | 15 | 52.4 | 0 | 31.6 | No |
| Slentz et al (16) | RCT | 48 | 49.5 | 26 | 30.4 | No |
| Slentz et al (16) | RCT | 52 | 49.7 | 30 | 30.5 | No |
| Sullivan et al (17) | RCT | 12 | 48.6 | 8 | 37.1 | No |
| Takahashi et al (37) | NA | 31 | 55.5 | 22 | 28.5 | No |
| Taniguchi et al (36) | RCT | 17 | 69 | NA | 23.1 | No |
| Ueno et al (11) | N-RCT | 15 | 39 | 7 | 31 | Yes |
| Vilar Gomez et al (15) | RCT | 30 | 49 | 14 | 31.5 | Yes |
| Vilar Gomez et al (15) | CaseCT | 87 | 51.9 | 0 | 29.2 | Yes |
| Yoshimura et al (22) | RCT | 12 | 61 | 11 | 27.3 | Yes |
| Zang et al (34) | RCT | 73 | 53.2 | 52 | 27.9 | No |
| Zang et al (34) | RCT | 73 | 54.4 | 51 | 28.1 | No |
| Zelber-Sagi et al (38) | RCT | 31 | 46.3 | 15 | 30.8 | No |

| Author (reference) | Protocol | Program | Session (min) | Frequency (days/week) | Program period (week) |
| --- | --- | --- | --- | --- | --- |
| Bacchi et al (19) | Aerobic | TRM, CY, or EM/CT | 60 | 3 | 16 |
| Bacchi et al (19) | Resistance | WMTR | NA | 3 | 16 |
| Bhat et al (18) | Aerobic | WLK, JOG or ARB | 30 | 5 | 24 |
| Cassidy et al (29) | Aerobic | ERG | NA | 3 | 12 |
| Chen et al (12) | Aerobic | ERG | 60 | 2 | 10 |
| Chen et al (12) | Aerobic | ERG | 60 | 2 | 10 |
| Cuthbertson et al (30) | Aerobic | TRM, ERG, EM/CT or rower | 30 | 3 | 16 |
| Fealy et al (28) | Aerobic | TRM | 60 | 7 | 1 |
| Hallsworth et al (25) | Aerobic and Resistance | ERG and BWTR | 35 | 3 | 12 |
| Haus et al (20) | Aerobic | TRM | 55 | 7 | 1 |
| Houghton et al (31) | Aerobic and Resistance | CY and WMTR | NA | 3 | 12 |
| Johnson et al (14) | Aerobic | ERG | 37.5 | 3 | 4 |
| Kantartzis et al (13) | Aerobic | WLK and/or SWM | NA | NA | 35 |
| Keating et al (24) | Aerobic | ERG and WLK | 37.5 | 3 | 8 |
| Keating et al (24) | Stretching | stretching, self-massage and Fit-ball program | 5 | 3 | 8 |
| Keating et al (24) | Aerobic | ERG and WLK | 52.5 | 4 | 8 |
| Keating et al (24) | Aerobic | ERG and WLK | 37.5 | 3 | 8 |
| Khaoshbaten et al (21) | Aerobic | NA | 30 | 3 | 12 |
| Lee et al (27) | Resistance | WMTR | 60 | 3 | 12 |
| Lee et al (27) | Aerobic | TRM | 60 | 3 | 12 |
| Oh et al (23) | Aerobic | WLK and/or JOG | 14.5 | 3 | 12 |
| Oh et al (23) | Aerobic | WLK and/or JOG | 30.9 | 3 | 12 |
| Pugh et al (35) | Aerobic | TRM and/or ERG | 30 | 3 | 16 |
| Rezende et al (32) | Aerobic | TRM | 30 | 2 | 24 |
| Shamsoddini et al (26) | Resistance | WMTR | 45 | 3 | 8 |
| Shamsoddini et al (26) | Aerobic | TRM | 45 | 3 | 8 |
| Shojaee-Moradie et al (33) | Aerobic and Resistance | NA | 20 | 4 | 16 |
| Slentz et al (16) | Aerobic | TRM, EM/CT, and/or ERM | 39 | 3 | 32 |
| Slentz et al (16) | Resistance | WMTR | 53 | 3 | 32 |
| Sullivan et al (17) | Aerobic | TRM | 44.8 | 5 | 16 |
| Takahashi et al (37) | Resistance | BWTR | 25 | 3 | 12 |
| Taniguchi et al (36) | Aerobic | ERG | 39 | 3 | 5 |
| Ueno et al (11) | Aerobic | WLK and/or JOG | 40 | 5 | 12 |
| Vilar Gomez et al (15) | Aerobic | JOG | 40 | 5 | 24 |
| Vilar Gomez et al (15) | Aerobic | JOG | 58.5 | 3 | 12 |
| Yoshimura et al (22) | Aerobic | Step exercises, ERG and WLK or JOG | 60 | 5 | 12 |
| Zang et al (34) | Aerobic | TRM and WLK | 30 | 5 | 48 |
| Zang et al (34) | Aerobic | WLK | 30 | 5 | 48 |
| Zelber-Sagi et al (38) | Resistance | WMTR | 40 | 3 | 12 |

| Author (reference) | dBMI | dALT | Evaluation modality | Significant improvement in steatosis | Significant weight loss | Changes in intrahepatic lipid (%) evaluated by 1HMR |
| --- | --- | --- | --- | --- | --- | --- |
| Bacchi et al (19) | -0.7 | 0.4 | ^1^HMR | Yes | Yes | -10 |
| Bacchi et al (19) | -0.6 | -5.33 | ^1^HMR | Yes | Yes | -12 |
| Bhat et al (18) | -1.3 | -46.5 | Bx | Yes | Yes | - |
| Cassidy et al (29) | NA | -6 | ^1^HMR | Yes | Yes | -2.7 |
| Chen et al (12) | -1.6 | -15.4 | US | Yes | No | - |
| Chen et al (12) | -0.4 | -3.3 | US | Yes | No | - |
| Cuthbertson et al (30) | -0.6 | -13 | ^1^HMR | Yes | Yes | -9.3 |
| Fealy et al (28) | 0.1 | -5.9 | ^1^HMR | No | No | -0.7 |
| Hallsworth et al (25) | -0.5 | -10 | ^1^HMR | Yes | Yes | -2.8 |
| Haus et al (20) | 0.1 | NA | ^1^HMR | Yes | No | -0.7 |
| Houghton et al (31) | 0 | -1 | ^1^HMR | Yes | No | -2 |
| Johnson et al (14) | -0.1 | -2.8 | ^1^HMR | Yes | No | -21 |
| Kantartzis et al (13) | -1.6 | -12.9 | ^1^HMR | Yes | Yes | -4.6 |
| Keating et al (24) | -0.5 | 1 | ^1^HMR | Yes | No | -2.4 |
| Keating et al (24) | 0.7 | -8 | ^1^HMR | No | No | 1.1 |
| Keating et al (24) | -0.5 | 3.5 | ^1^HMR | Yes | No | -2.5 |
| Keating et al (24) | 0.2 | -0.3 | ^1^HMR | Yes | No | -0.8 |
| Khaoshbaten et al (21) | -0.9 | -17.6 | US | Yes | Yes | - |
| Lee et al (27) | -0.6 | NA | ^1^HMR | Yes | Yes | -2 |
| Lee et al (27) | -0.3 | NA | ^1^HMR | Yes | Yes | -1.9 |
| Oh et al (23) | -1.9 | -8.2 | CAP | Yes | Yes | - |
| Oh et al (23) | -3.4 | -13.5 | CAP | Yes | Yes | - |
| Pugh et al (35) | -1 | -41 | ^1^HMR | Yes | No | -20.6 |
| Rezende et al (32) | -0.55 | -7.1 | CAP | No | No | - |
| Shamsoddini et al (26) | -0.7 | -14.7 | US | Yes | Yes | - |
| Shamsoddini et al (26) | 0.6 | -12.5 | US | Yes | No | - |
| Shojaee-Moradie et al (33) | -1.1 | -14.3 | ^1^HMR | Yes | Yes | -9.7 |
| Slentz et al (16) | NA | -4.3 | CT | Yes | Yes | - |
| Slentz et al (16) | 0.7 | -2.8 | CT | No | Yes | - |
| Sullivan et al (17) | 0 | -6.3 | ^1^HMR | Yes | No | -10.3 |
| Takahashi et al (37) | -0.1 | -18.8 | US | Yes | No | - |
| Taniguchi et al (36) | -0.1 | -2 | ^1^HMR | Yes | No | -0.6 |
| Ueno et al (11) | -3 | -56 | Bx | Yes | Yes | - |
| Vilar Gomez et al (15) | -3.5 | -19 | Bx | Yes | Yes | - |
| Vilar Gomez et al (15) | -3.7 | -11.7 | CAP | Yes | Yes | - |
| Yoshimura et al (22) | -2 | NA | CT | Yes | Yes | - |
| Zang et al (34) | -1.4 | -2.7 | ^1^HMR | Yes | Yes | -6.3 |
| Zang et al (34) | -1 | -2.2 | ^1^HMR | Yes | Yes | -5.9 |
| Zelber-Sagi et al (38) | -0.1 | -5.3 | US | Yes | No | - |

ARB, rhythmic aerobic exercises; BWTR, bodyweight training without machine; Bx, biopsy; CAP, controlled attenuation parameter; CaseCT, case-control study; CT, computed tomography; CY, cycling; EM/CT, elliptical machine or cross trainer; ERG, Ergometer; JOG, jogging; NA, not available; N-RCT, non- randomized controlled trial; RCT, randomized controlled trial; SAT, single-arm trial; SWM, swimming; TRM, treadmill; US, ultrasonography; WLK, brisk walking or walking; WMTR, weight training with machine; ^1^HMR ^1^H magnetic resonance.
