# Supplementary material for: Therapeutic Approaches to Nonalcoholic Fatty Liver Disease: Exercise Intervention and Related Mechanisms
Source: Front Endocrinol (Lausanne). 2018 Oct 15;9:588. doi: 10.3389/fendo.2018.00588 (PMC6196235; doi:10.3389/fendo.2018.00588)
Supplement: Supplementary file 2 [file Table_2.DOCX]

Supplementary 2. Aerobic training protocols for nonalcoholic fatty liver disease: literature review.

|  | Effective on steatosis | Ineffective on steatosis | p value |
| --- | --- | --- | --- |
| Number | 35 | 4 |  |
| Duration (min/session) | 39 (14.5-60)* | 41.5 (5-60) | 0.593 |
| Frequency (day/week) | 3 (2-7)** | 3 (2-7) | 0.733 |
| Period (week) | 12 (1-48) | 16 (1-32) | 0.861 |
| Total training time (h) | 24 (6.4-120)* | 15.5 (2-84.8) | 0.725 |

*n=31 and **n=34. Data is shown in median (range).
