# Supplementary material for: Therapeutic Approaches to Nonalcoholic Fatty Liver Disease: Exercise Intervention and Related Mechanisms
Source: Front Endocrinol (Lausanne). 2018 Oct 15;9:588. doi: 10.3389/fendo.2018.00588 (PMC6196235; doi:10.3389/fendo.2018.00588)
Supplement: Supplementary file 3 [file Table_3.docx]

Supplementary material 3. Ineffective protocols of exercise training on liver steatosis of nonalcoholic fatty liver disease: literature review.

| Training | Diet consultation | Duration  (min/session) | frequency (days/week) | Period  (week) | Age  (year) | Gender (female%) | Mean BW change (kg) | Reference |
| --- | --- | --- | --- | --- | --- | --- | --- | --- |
| Stretching | No | 5 | 3 | 8 | 39.1 | 75 | -0.7 | 24 |
| Aerobic | No | 30 | 2 | 24 | 56.2 | 100 | +0.6 | 32 |
| Aerobic | No | 60 | 7 | 1 | 58 | NA | -0.1 | 28 |
| Resistance | No | 53 | 3 | 32 | 49.7 | 57.7 | -0.7 | 16 |

BW, body weight; NA, not available.
